# 3D hindlimb joint mobility of the stem-archosaur *Euparkeria capensis* with implications for postural evolution within Archosauria

**DOI:** 10.1038/s41598-020-70175-y

**Published:** 2020-09-21

**Authors:** Oliver E. Demuth, Emily J. Rayfield, John R. Hutchinson

**Affiliations:** 1 School of Earth Sciences, University of Bristol, Wills Memorial Building, Queens Road, Bristol, BS8 1RJ UK; 2 Structure and Motion Laboratory, Department of Comparative Biomedical Sciences, The Royal Veterinary College, Hawkshead Lane, Hatfield, AL9 7TA UK

**Keywords:** Palaeontology, Biomechanics

## Abstract

Triassic archosaurs and stem-archosaurs show a remarkable disparity in their ankle and pelvis morphologies. However, the implications of these different morphologies for specific functions are still poorly understood. Here, we present the first quantitative analysis into the locomotor abilities of a stem-archosaur applying 3D modelling techniques. μCT scans of multiple specimens of *Euparkeria capensis* enabled the reconstruction and three-dimensional articulation of the hindlimb. The joint mobility of the hindlimb was quantified in 3D to address previous qualitative hypotheses regarding the stance of *Euparkeria*. Our range of motion analysis implies the potential for an erect posture, consistent with the hip morphology, allowing the femur to be fully adducted to position the feet beneath the body. A fully sprawling pose appears unlikely but a wide range of hip abduction remained feasible—the hip appears quite mobile. The oblique mesotarsal ankle joint in *Euparkeria* implies, however, a more abducted hindlimb. This is consistent with a mosaic of ancestral and derived osteological characters in the hindlimb, and might suggest a moderately adducted posture for *Euparkeria*. Our results support a single origin of a pillar-erect hip morphology, ancestral to Eucrocopoda that preceded later development of a hinge-like ankle joint and a more erect hindlimb posture.

## Introduction

**Figure 1:**
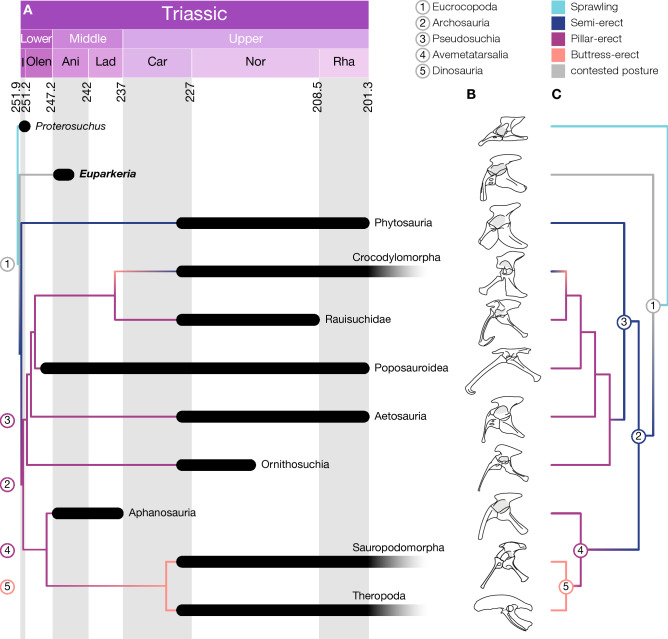
Pelvic morphology and evolution of hip joint articular morphology within archosaurs. A, time-calibrated phylogenetic tree based on the Nesbitt et al.^61^ tree shows the rapid diversification of archosaurs in the Lower Triassic. B, pelvic girdles of various stem-archosaurs and archosaurs. C, evolution of hip joint articular morphology based on the Ezcurra^36^ topology. Note the difference in the inferred ancestral posture for Archosauria and Pseudosuchia depending on the tree topology. Depicted taxa from top to bottom: *Proterosuchus*, redrawn from Ezcurra et al.^82^, *Euparkeria*, based on SAM PK 5867 and SAM PK 6047A, Phytosauria: *Parasuchus*, redrawn from Chatterjee^83^, Crocodylomorpha: *Alligator*, redrawn from Romer^84^, Rauisuchidae: *Postosuchus*, redrawn from Chatterjee^85^, Poposauroidea: *Poposaurus*, redrawn from Schachner, Manning & Dodson^86^, Aetosauria: *Stagonolepis*, redrawn from Walker^87^, Ornithosuchia: *Ornithosuchus*, redrawn from Sereno^2^, Aphanosauria: *Teleocrater*, redrawn from Nesbitt et al.^38^, Sauropodomorpha: *Plateosaurus*, based on specimen GPIT1, Theropoda: *Apteryx*, redrawn from Schachner, Manning & Dodson^86^.

Archosaurs were the predominant group of large terrestrial and aerial vertebrates in the Mesozoic era and included pterosaurs, the familiar dinosaurs (including birds), crocodylomorphs and an intriguing variety of Triassic forms. Their well-documented fossil record allows retracing the evolution of the dramatic differences in morphology and locomotion in this group. By the Middle Triassic, the clade had already reached high levels of morphological and functional disparity, especially in the hip and ankle joints^1–5^ (Fig. 1), but this disparity was subsequently lost in the following mass extinctions. Birds and crocodiles are two morphological and ecological extremes and the only surviving groups. Separated by ~ 250 million years of evolutionary history, they show major differences in posture, stance and gait^6,7^. The assignment of postural ‘grades’ within archosauriforms and basal archosaurs as well as investigation into their locomotion has almost exclusively been based on qualitative assessments. Different hypotheses have been proposed either through assessing the osteology of the pelvis and hindlimb^1,8–10^, comparison with extant analogues^11^ or the interpretation of trackways^12,13^. Quantitative biomechanical analyses of the locomotion of basal archosaurs and archosauriforms have, however, been mostly neglected and only rarely assessed^10,14^. Generally, quantitative biomechanical approaches have overwhelmingly focused on non-avian dinosaurs (e.g.^15–27^); however, to comprehend the evolution of the different morphologies and the associated locomotor specialisations within archosaurs, it is essential to assess what the ancestral condition for Archosauria was.

**Figure 2:**
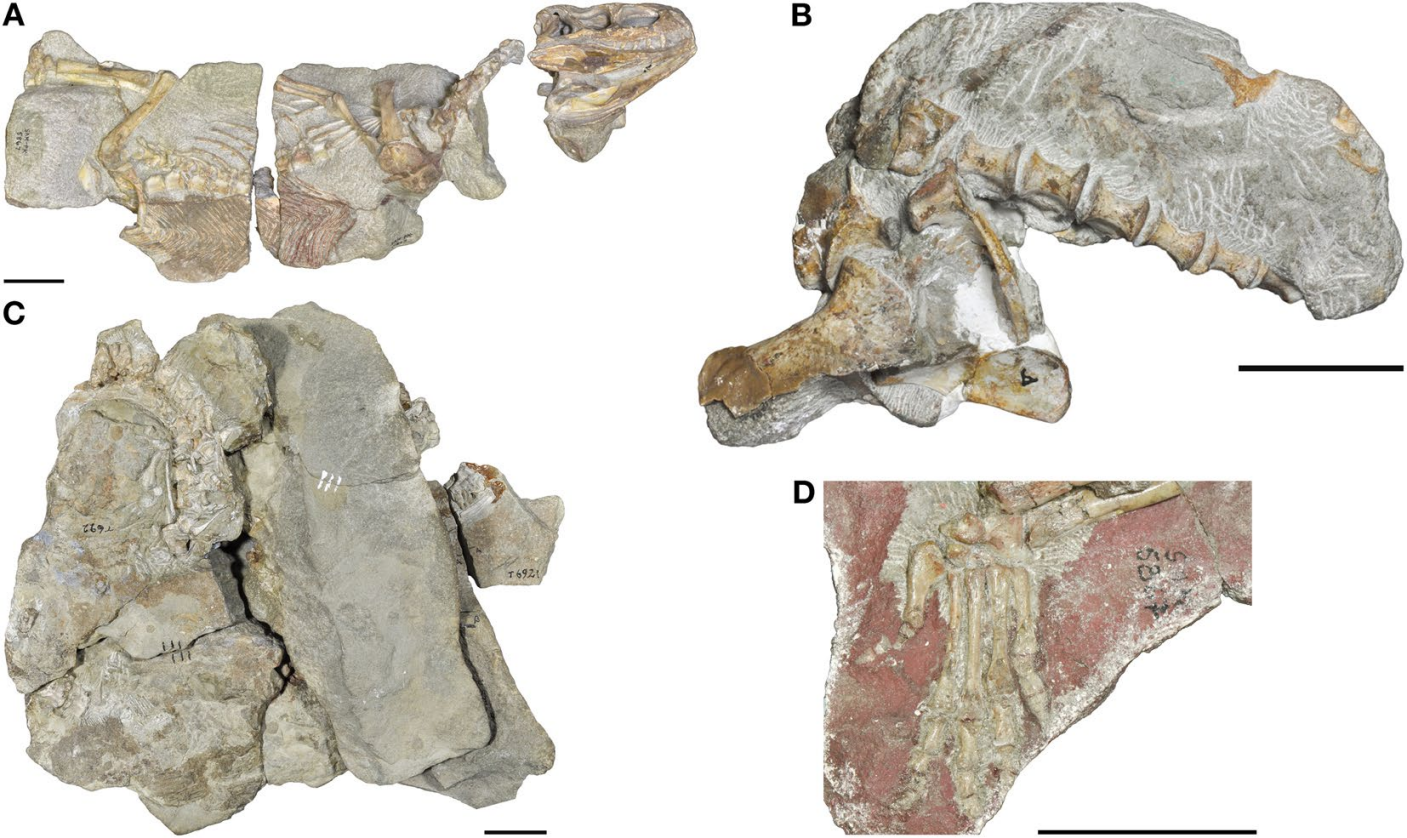
μCT-scanned specimens of *Euparkeria capensis*. **(A)** Holotype SAM PK 5867. **(B)** pelvis block of SAM PK 6047A. **(C)** Articulated blocks of UMZC T.692. **(D)** Articulated foot of SAM PK K8309. Images courtesy of R. Butler and R. Sookias. Scale bars represent 3 cm.

*Euparkeria capensis* is a small eucrocopodan archosauriform from the early Middle Triassic (early Anisian) Burgersdorp Formation near Aliwal North, Eastern Cape, South Africa; and only from a single horizon therein, the Subzone B of the *Cynognathus* Assemblage Zone^28,29^. Its osteology is well known from numerous well-preserved specimens^29,30^ (Fig. 2); however, it still lacks a comprehensive and thorough monographic redescription. *Euparkeria* has been recovered close to the origin of Archosauria in many phylogenetic analyses^2, 31–38^ . Furthermore, *Euparkeria* appears to morphologically and ecologically resemble the expected ancestor of Archosauria^4,31^ and thus it is an ideal study subject for assessing the ancestral locomotory capabilities of archosaurs. Several hypotheses regarding the stance and gait of *Euparkeria* have been proposed, ranging from a ‘semi-erect’ posture during locomotion^1,9,39^ to a more widely accepted sprawling interpretation^10,40,41^. Based on limb proportions *Euparkeria* was suggested to have been capable of facultative bipedal locomotion^40,42^, although this has been questioned^39,41^. Even the foot posture is controversial and is either interpreted as being digitigrade^40,43^ or plantigrade^1,8,9,11^. Ultimately, none of these hypotheses have been quantitatively assessed; and essentially all are based on almost two-dimensional visual interpretations of specimens still encased in matrix, rather than 3D manipulation of prepared or digital specimens.

The articulation and functional morphology of the ankle joint of *Euparkeria* has been thoroughly examined and described in great detail^1,2,11,33,43–45^, however the exact three-dimensional (3D) articulation of the bones within the crus, distal tarsals and metatarsals has never been fully assessed, mostly due to the small size of the bones and the fact that most of them are still partially embedded in matrix (Fig. 2). Likewise, the pelvic girdle has only been described based on partially exposed elements from multiple specimens^40^.

Here, we used 3D models, derived from μCT-scans of several specimens, to accurately reconstruct the 3D morphology and articulation of the pelvic girdle and ankle of *Euparkeria* in order to investigate its functional morphology and locomotory capabilities. While the osteological range of motion (ROM) of limb joints has been previously quantified in Devonian and Permian tetrapods^46–48^, there have been no previous attempts to quantify hip joint mobility, or the osteological ROM of any other joint, in Triassic archosaurs (see ^10^). We hope that the application of quantitative computational methods to stem archosaurs could lead to new insights into the evolution of their functional morphology and hindlimb biomechanics. In particular, the combination of independent lines of evidence (e.g. see ^48^) allows for a more accurate reconstruction of the potential postures of extinct taxa. We quantified the ROM to determine the maximal joint excursion in the hip joint and compared it with the ankle joint rotation axes, thereby testing whether previous qualitative assessments of hindlimb posture were supported through a quantitative biomechanical analysis. We then used these results to revisit the longstanding question of whether stem-archosaurs were more sprawling or capable of a more adducted posture^1,8,10,12,49,50^.

## Results

### 3D reconstruction of the pelvic girdle

**Figure 3:**
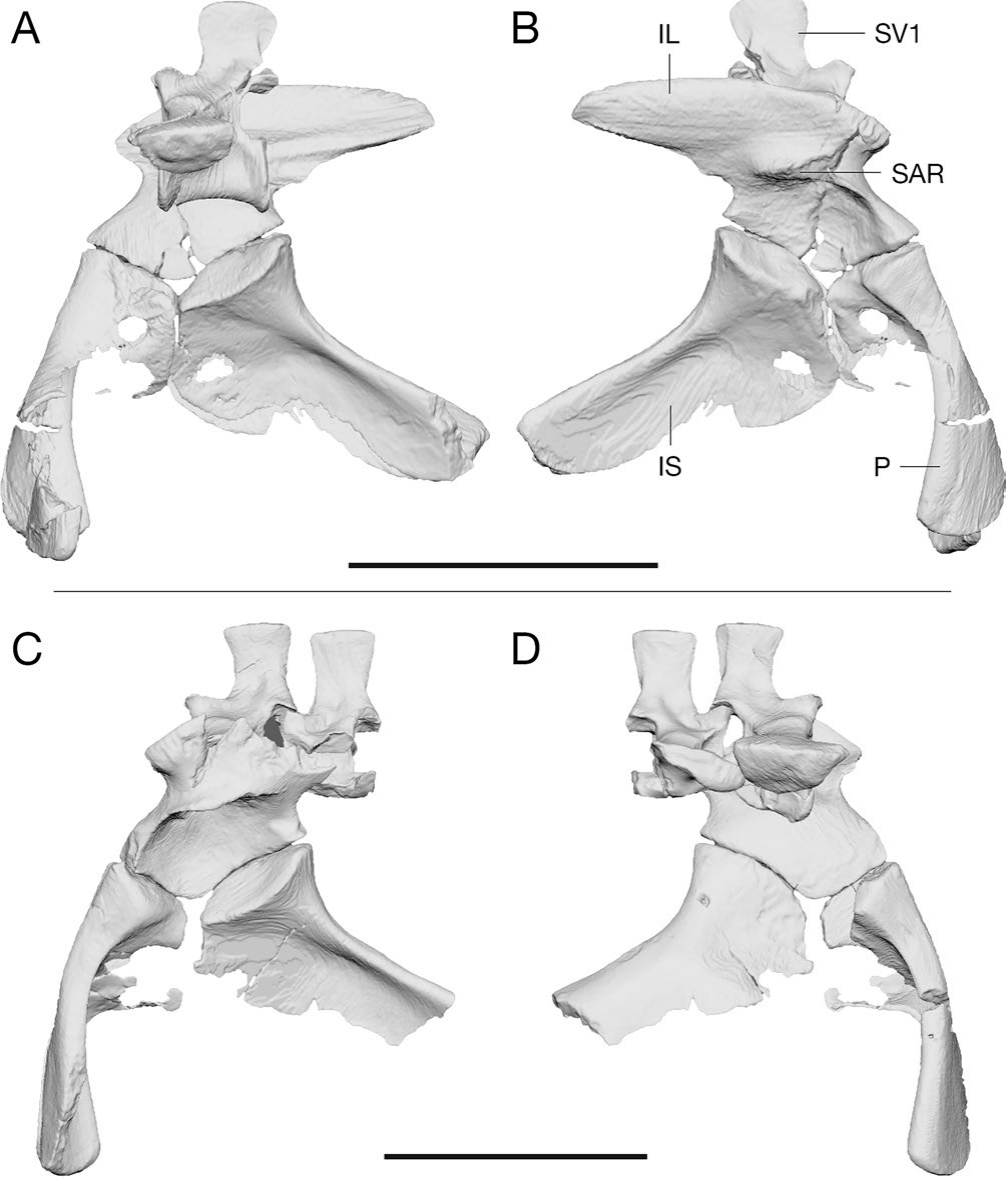
Comparison of the segmented *Euparkeria* pelves. **(A)** Rearticulated pelvis of SAM PK 5867 in left lateral view. **(B)** In right lateral view. **(C)** Rearticulated pelvis of SAM PK 6047A in left lateral view. **(D)** In right lateral view. The second sacral vertebra in SAM PK 5867 is preserved, however both sacral ribs were lost due to erosion and could therefore not provide additional information for the articulation of the pelvis and that region therefore was not segmented. *IL* ilium, *IS* ischium, *P* pubis, *SAR* supra-acetabular rim, *SV1* sacral vertebra 1. Scale bars are 3 cm.

The pelvis is preserved in two of the studied specimens: in the holotype of *Euparkeria*, SAM PK 5867, and in SAM PK 6047A. SAM PK 5867 is still partially covered in matrix and the femur is overlaying most of the right ilium, thus obscuring most of the pelvic bones. However, the μCT scans revealed that the pelvis is nearly complete, missing only the left ilium, the distal tip of the left ischium, most of the left pubis and the ribs of the second sacral vertebra (Fig. 3A,B). The pelvis has been somewhat crushed during fossilisation and the bones have thus moved slightly out of articulation, however most bones remain in immediate association. The original 3D shape of the bones is generally well preserved, with only minor alterations due to cracks running through the ilium and right pubis. However, the positioning of the femur on top of the ilium taphonomically distorted the shape of supra-acetabular rim, leaving it less pronounced than in the second specimen.

In SAM PK 6047A the pelvic girdle was disarticulated prior to burial and is only partially complete (Fig. 3C,D). The preserved bones include a partial left ilium, missing the postacetabular process, the mostly complete left and right pubes, the left ischium, missing the distal part of the shaft, and both sacral vertebrae, however both of which have lost the left sacral rib due to erosion. Fortunately, the disarticulation of the specimen and the positioning of the pelvic bones during fossilisation protected the supra-acetabular rim from deformation, unlike in the holotype. It is thus apparent that the supra-acetabular rim is well developed and covers a relatively deep acetabulum. The inclined ilia, due to the ventrolaterally projecting sacral ribs, further emphasize the prominence of the supra-acetabular rim and result in an acetabulum that opens subventrally, which allows for the complete coverage of the femoral head.

### Hip joint spacing and range of motion

In total 16 simulations were set up and 14 of those were run for the hip ROM analysis to quantify the ROM and test the dependency of the ROM on the different joint setups (see Supplementary Information). The label of each simulation is composed of four components, which indicate its setup depending on the different simulation parameters. The first component is the specimen number, referencing the pelvis used for the simulation, the second and third indicate the primitive shape fitted to the acetabulum and the femoral head respectively, finally the number represents the amount of additional joint spacing as a percentage of femoral length for potential epiphyseal cartilage. For example, simulation SAM PK 6047A SE0 was performed based on the segmented pelvis of SAM PK 6047A, with the joint centre derived from the fitted sphere to the acetabulum (S), the fitted ellipsoid to the femoral head (E) and 0% of additional epiphyseal cartilage (0). The osteological ROM of *Euparkeria* varied dramatically depending on the geometric primitive shape used to determine the joint centres.

The joint spacing, defined as the average distance between the femoral head and acetabulum, depended on the fitted geometric primitive shape and the amount of additional epiphyseal cartilage. It ranged from − 1.605 to 8.486 mm (Supplementary Table S4). Overall, the distances between the articular surfaces of the femur and acetabulum were similar between both specimens. The joint spacing was slightly larger for the fitted sphere in SAM PK 5867 than in SAM PK 6047 (difference 0.353 mm) and slightly smaller for the fitted ellipsoid (difference 0.143 mm). This was due to the fact that the supra-acetabular rim is damaged in SAM PK 5867 and the acetabulum is slightly shallower, which resulted in a larger radius of the fitted sphere and smaller radii for the fitted ellipsoid.

**Figure 4:**
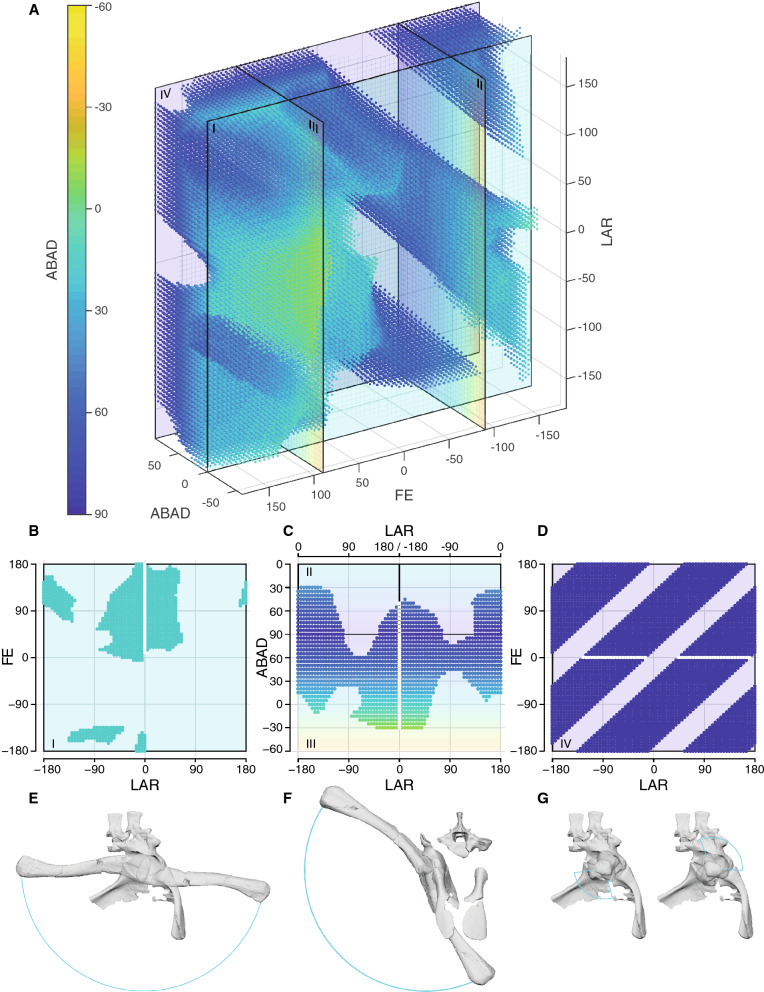
Hip joint ROM analysis. **(A)** Point cloud of all viable poses for simulation SAM PK 6047A SE0. **(B–D)** ROM cross-sections; **(B)** ABAD = 0° (I); **(C)** FE = − 90° (II) and FE = 90° (III); **(D)** ABAD = 90° (IV); roman numerals indicate position of cross-sections in **(A)**. The white lines indicate the path of the femur shown in (**E**, **F)**. To show the full ABAD swing in **(C)** two cross-sections (II and III) were combined into a single plot. **(E–G)** Corresponding maximal excursions of the femur to the cross-sections above in lateral view **(E, G)** and cranial view **(F)**. Note the disconnected ‘locked’ areas of viable poses **(D)** indicating that the femur cannot swing through the stance phase **(G)**.

We deemed simulation setup SAM PK 6047A SE0 (Fig. 4A; i.e. sphere fit to acetabulum, ellipsoid fit to femoral head, no added cartilage) the most likely based on comparison with Nile crocodile (*Crocodylus niloticus*) hip joint spacing derived from CT scans (Supplementary Information and Supplementary Tables S3–S4). Furthermore, specimen SAM PK 6047A is better preserved, as the supra-acetabular rim is complete in this specimen and the acetabulum is not distorted, therefore simulations based on this specimen are more reliable. The taphonomic deformation of SAM PK 5867 resulted in an overly wide fit for the fitted sphere and thus a rotation centre further away from the acetabulum. Thus, in SAM PK 5867 the poses of the femur were not restricted in either long-axis rotation (LAR) or flexion/extension (FE) when the femur was positioned sub-horizontally (Supplementary Fig. S1). In contrast, these poses were heavily restricted in the simulations using the better-preserved ilium of SAM PK 6047A—in particular, LAR and FE were ‘locked’ in two separate clusters. Due to this ‘locking’, the LAR was heavily restricted and the femur could not reach from one cluster into the other at abduction/adduction (ABAD) > 75° (Fig. 4D,G; Supplementary Video S1). The movement is blocked by the supra-acetabular rim colliding with the femoral head and is not an artefact of apparently disconnected joint spaces but a result of the hip morphology. With decreasing ABAD values this ‘locking’ disappears. To allow the femur to rotate freely in LAR, unaffected by the supra-acetabular rim, it needs to be adducted by at least 15° from the horizontal plane. This osteological limitation on the LAR is in contrast to crocodylians or more sprawling taxa, e.g. *Iguana,* where no such hard-tissue limitations are present^46,51^, although more directly comparable datasets are needed.

Overall, simulation SAM PK 6047A SE0 resulted in 58,776 osteologically viable poses with a volume of 6,669,600°^3^. It appears that *Euparkeria* was osteologically able to adduct the hindlimb into a vertical posture or even surpass the midline (‘hyper-adduct’) (Fig. 4C,F), while large ranges of less-adducted poses remained feasible.

### Articulation of the ankle joint

**Figure 5:**
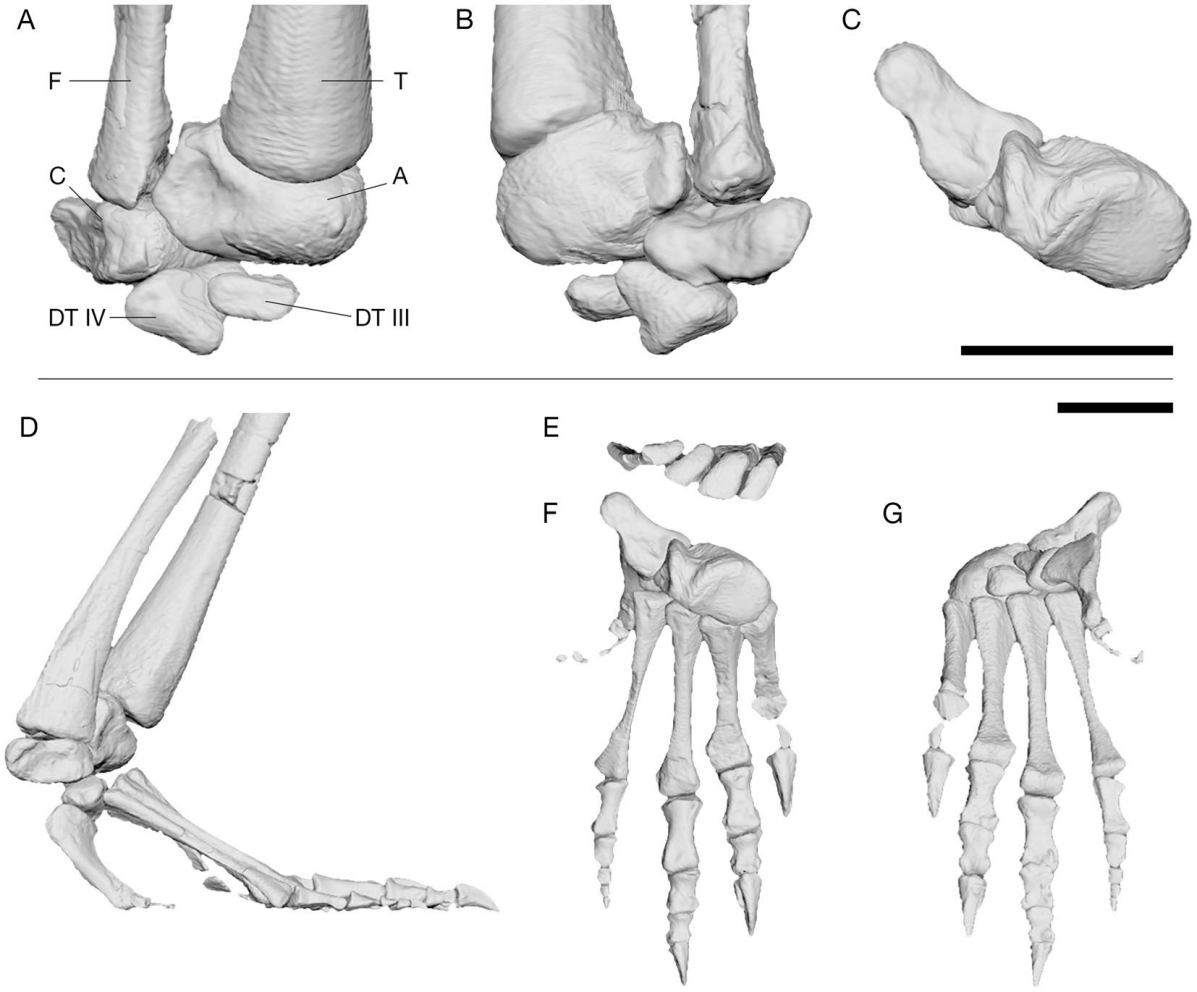
Composite three-dimensionally articulated right foot and ankle of *Euparkeria*, based on SAM PK 5867, SAM PK K8309 and UMZC T.692. **(A)** Tarsus in cranial/dorsal view. **(B)** Caudal/ventral view of tarsus. **(C)** Articulation of astragalus and calcaneus in proximal view. **(D)** Reconstructed pes in lateral view. **(E)** Articulation of metatarsals I–IV in proximal view. **(F)** pes in dorsal view. **(G)** pes in ventral view. *A* astragalus, *C* calcaneum, *DT III* distal tarsal III, *DT IV* distal tarsal IV, *F* fibula, *T* tibia. Scale bars represent 2 cm.

The bones obtained from the µCT scans allowed virtual reassembly of the foot and ankle skeleton in its estimated natural posture (Fig. 5). The fibular facet on the calcaneum is a shallow convex depression on its dorsal surface^44,45^. Sullivan^45^ suggested that this facet might have allowed for limited movement against the fibula during flexion and extension of the ankle joint. However, based on the novel 3D articulation of the proximal and distal ankle joints presented herein, this motion appears unlikely. The astragalo-calcaneal joint is hinge-like with an craniocaudal groove on the astragalus and a concave articular surface on the calcaneum, thus rotatory movement of the calcaneum around the astragalus and fibula (flexion–extension) appears minimal (unlike in ^1^). We suggest that the fibulo-calcaneal joint instead assisted rotational movement of the fibula around the ankle joint (pronation and supination/LAR), in order to keep the foot steady on the ground as part of a non-parasagittal gait.

The subtriangular distal tarsal (DT) IV possesses a caudally projecting spur, which articulated with the astragalus dorsally and DT III medially and is slightly concave laterally for the articulation with the articular convexity of the calcaneum (Fig. 5B). The articular surface for the L-shaped metatarsal (MT) V is angled lateroventrally, which results in a ventrally projecting MT V (Fig. 5). Craniomedally it forms a relatively flat, sub-triangular surface for articulation with the MT IV. The proximal articular faces of MT III and IV are slightly concave to allow rotation around the flat articular surfaces of the distal tarsals, whereas those of MT I and II are flat and articulate with the roller surface of the astragalus. Metatarsals I to IV are closely packed proximally, with considerable overlap of their proximal ends (Fig. 5E).

**Figure 6:**
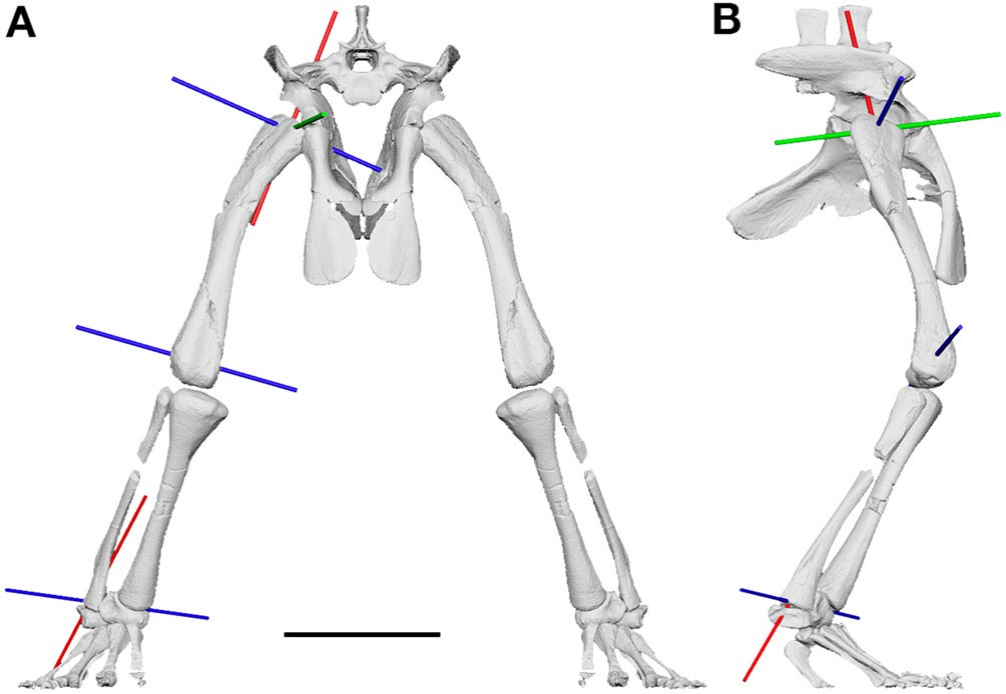
Most adducted hindlimb posture of *Euparkeria*. **(A)** Cranial view. **(B)** Right lateral view. Note the posture is not fully adducted, to accommodate the oblique angle of the ankle joint. Less hip abduction or further extension of the limb would result in the medial inclination of the foot. The rotation axes of the hindlimb are coloured depending on the possible motion: flexion/extension (blue), long-axis rotation (red) and abduction/adduction (green). Scale bar represents 3 cm. Note the non-alignment of the flexion/extension axes, making a fully parasagittal gait impossible.

The main rotation axis of the mesotarsal ankle joint in *Euparkeria* is oblique to the knee joint and further oblique to the orientation of the metatarsophalangeal joints (Supplementary Table S5). The rotation of the foot around the ankle joint therefore resulted in a medially inclined foot in relation to the crus during flexion and extension (Supplementary Video S2–S3). For the most vertically-aligned pose possible with the foot still firmly on the substrate (Fig. 6), it is evident that a parasagittal gait was not possible (Supplementary Information). The femur needed to be rotated about − 6° around its long axis (internal rotation), abducted by 25° and extended by 77° in relation to the reference pose of (0°/0°/0°) (see^52,53^; Supplementary Information). While this is the position with the most vertically-aligned hindlimb posture (most adduction) that *Euparkeria* theoretically could have assumed, e.g. potentially during mid-stance of locomotion or during standing, there are many poses with less adduction that *Euparkeria* could have assumed in life. Osteological/ROM data alone cannot discern which of these is more plausible; we present the most adducted pose to illustrate the non-alignment of the joint axes even in extreme poses (Fig. 6; Supplementary Information).

## Discussion

Our first quantitative assessment of the ROM in a stem archosaur was enabled by a sophisticated and robust simulation setup in Autodesk Maya. We showed that in *Euparkeria* the osteology permitted the adduction of the femur into a fully vertical position (FE = 90°, ABAD = 0°), and it was even feasible to further adduct the femur medially and overstep the other limb (ABAD = − 30°; Supplementary Video S1). While osteologically possible, the latter extreme pose almost certainly was not achievable in vivo, as soft tissue would have restricted such excessive excursion of the limb^53^. A wide variety of less adducted postures, ranging up until 35° above the horizontal plane (FE = − 90°, LAR = − 180°/180°; Fig. 4), were feasible, thus the ROM analysis was unable to rule out any less adducted postures for *Euparkeria*. However, the osteological restriction on long-axis rotation (− 10° to 40°) in sub-horizontal femur poses indicated that *Euparkeria* was potentially unable to engage in fully sprawling gaits which rely on external (positive) LAR of the femur^51,54^, e.g. in late swing and early stance, and/or use high degrees of femoral abduction (Supplementary Figure S9) and thus may indicate a more adducted hindlimb posture for *Euparkeria* (see^48^). Additionally, this osteological limitation on LAR is absent in known taxa capable of a more sprawling gait, e.g. salamanders, *Iguana* or crocodylians^46,51^, and potentially further implies a less abducted limb during locomotion for *Euparkeria* (unlike in^10^). However, further studies of 3D ROM envelopes in more sprawling extant taxa; e.g. salamanders, varanids and iguanas; and the relationship between osteological and in vivo ROM in such taxa, are necessary to further characterize sprawling motion and its musculoskeletal constraints in order to draw more detailed inferences about locomotion in extinct taxa. While many poses (both sprawling and erect) could not be excluded for *Euparkeria* based on the osteological ROM alone, we caution against drawing conclusions directly based on these osteologically valid poses, especially those close to the border of the ROM map (Fig. 4). Some of these poses might still not have been possible in vivo, as the osteological ROM usually overestimates joint mobility and only a subset is actively used during locomotion^51,53,55^. Further, we caution against comparing osteologically valid poses directly with extant taxa, as such comparisons are difficult and potentially flawed^56^, especially when they are based on a different 3D reference frame; see^57^. While many sprawling postures could not directly be excluded, the hip joint morphology differs substantially from any extant taxa with a more sprawling gait, therefore direct inferences are not warranted and such postures are potentially less likely.

Our approach, combining 3D articulation and ROM analyses, demonstrates conflicting patterns in the hip and ankle joint. *Euparkeria* exhibits several derived traits indicating a more erect hindlimb posture while also retaining ancestral characters consistent with a less erect posture. Notably the hip, with the deep acetabulum and supra-acetabular rim, opens the possibility for a fairly adducted femoral orientation, with a ‘pillar-erect’ support of the body^32,58^, which was further supported by the ROM analysis, showing that high degrees of hip adduction were feasible. However, the ankle structure, with the craniomedially inclined tibia and the oblique angle of the mesotarsal joint, points strongly towards a more abducted hindlimb^1^. Furthermore, our ROM analysis of the hip left open a wide functional space that did not conclusively exclude some degree of sprawling; interpretations of our results may vary. Overall, *Euparkeria* demonstrates a mixture of ancestral and derived morphological traits, which is also reflected in the simulation results, indicating that the classical trend towards more adducted postures in archosaurs was rather complex, and less straightforward than previously thought^1,8,10,13^. While *Euparkeria* had the ability in the hip joint to assume a fully adducted and ‘pillar-erect’ articulation, it probably did not adopt a fully adducted posture during locomotion, as its ankle structure does not seem suitable for a parasagittal gait. Consequently, a posture with moderate femoral adduction (neither fully sprawling nor fully erect, but somewhere in-between; and yet non-crocodylian; see^9,10,59^) for *Euparkeria* is in our view the most reasonable interpretation, although many postures fitting this description remain feasible in our results. Further resolution of the posture and gait of *Euparkeria* or other stem-archosaurs would require biomechanical methods that address how soft tissues such as muscles and the nervous system might have controlled hindlimb function.

**Figure 7:**
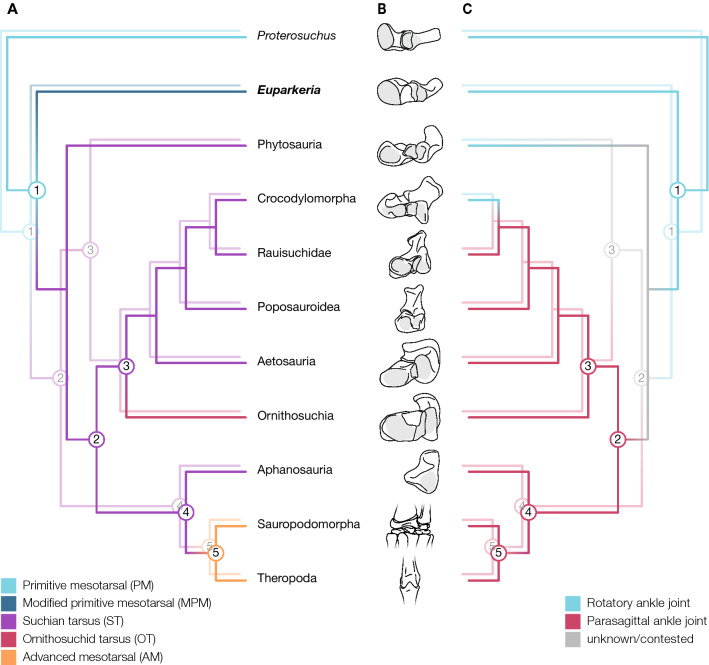
Ankle evolution within Archosauriformes. **(A)** Evolution of different ankle morphologies for both the Nesbitt^61^ (foreground) and Ezcurra^36^ (shadowed) topologies. The suchian tarsus evolved once and is ancestral to phytosaurs, pseudosuchians and avemetatarsalians in either tree. The nomenclature of the ankle morphotypes follows Sullivan^45^. **(B)** Illustrations of ankle morphologies for exemplary taxa of the displayed groups. **(C)** Uncertain timing of the transition between a rotary and parasagittal ankle joint within archosaurs. Phytosaurs could either represent a transitional phase^61^ (in the foreground) or either a reversal of the joint function or a parasagittal ankle joint evolved twice independently^36^ (shadowed). The ankle bones of the taxa as in Fig. 1 are depicted.

Our new insights into the hip structure in *Euparkeria* support a single origin of a pillar-erect hip morphology in both the Nesbitt^35^ and Ezcurra^36^ phylogenetic tree topologies, meaning that this articular morphology can be regarded as ancestral to at least Eucrocopoda, contrasting with previous hypotheses^1,10,60^. As we have cautioned, however, such a morphology does not necessarily suggest a ‘fully erect’ limb posture nor necessarily allow a parasagittal gait. The ancestral locomotor stance in archosaurs can thus not be inferred from the hip morphology alone. While we infer that the pillar-erect hip morphology arose prior to Archosauria (unlike in^10,60^), an ankle joint permitting a parasagittal gait appeared later on. Indicators of a pillar-erect hip morphology are laterally inclined ilia, due to the ventrolaterally projecting sacral ribs and a pronounced supra-acetabular rim, therefore allowing the ilium to completely cover the femoral head. This condition appears to be present in the basal avemetatarsallian *Teleocrater rhadinus*^38,61^ (ilium NHMUK PV R.6795 and second sacral vertebra NMT RB519) and the Triassic pseudosuchian ornithosuchids, e.g. *Riojasuchus tenuisceps*^62,63^. It thus seems likely that the pillar-erect hip morphology evolved as a precursor to the ‘buttress-erect’ morphology of both dinosauriforms^60,64^ and early crocodylomorphs^10,32,65^, and originated once at the base of Eucrocopoda. We infer that phytosaurs and extant crocodylians lost the erect hip morphology of their ancestors; pillar-erect in the former and buttress-erect in the latter; facilitating a more abducted hindlimb posture as a secondarily derived adaptation for their amphibious lifestyle. This is consistent with previous interpretations of the condition in crocodylians^9,10,66^ but novel in terms of phytosaurs (unlike in ^10^); thus phytosaurs show an additional level of convergent evolution with crocodylians. Depending on the interpretation of phytosaurs as either early-diverging pseudosuchians^34,36^ or stem-archosaurs^30,35,37,61^, the suchian tarsus might have evolved within Archosauria, or just outside this clade (Fig. 7A). While the tarsus of phytosaurs is morphologically similar to other suchian tarsi (Fig. 7B), functionally it is more similar to the ankle joint in *Euparkeria*, having an oblique flexion–extension axis^1^. This is either part of the transition from less erect stem-archosaurs towards an ankle joint permitting a more parasagittal gait, as seen in more derived archosaurs, or a reversal linked to the semi-aquatic adaptations in phytosaurs (Fig. 7C), as in extant crocodylians^9^. However, the phylogenetic position of phytosaurs needs to be settled and the functional evolution of their ankle joints quantitatively assessed before any further conclusions regarding the ancestral locomotion type of Archosauria can be drawn. Regardless, we speculate that the pillar-erect hip morphology ancestral to Archosauria released evolutionary constraints on the ankle joint within Archosauria (perhaps rendering obsolete the oblique flexion–extension axis of the ankle joint to permit a permanent contact of the foot with the substrate), later functionally facilitating the evolution of a hinge-like ankle joint with approximately a single degree of freedom suitable for a parasagittal gait in both pseudosuchians (suchian and ornithosuchid tarsus) and ornithodirans (advanced mesotarsal ankle joint^43^) and thus a fully adducted posture. These changes then further enabled the evolution of a bipedal locomotion and/or a digitigrade stance in poposaurids^67–69^ and dinosauromorphs^64,70,71^.

In conclusion, we add further evidence to the homoplastic manner of postural evolution within archosaurs (see^10^). We caution that the hindlimb posture of stem-archosaurs cannot be determined relying solely on qualitative morphological characters (especially single traits; e.g. hip articulations), and different lines of evidence need to be combined for conclusive results. While *Euparkeria* constrains the origin of the pillar-erect hip morphology to the base of Eucrocopoda, the ankle structure enabling a highly adducted hindlimb posture with a parasagittal gait evolved later on within Archosauria, thus a pillar-erect hip morphology does not necessarily warrant a fully adducted ‘pillar-erect’ posture.

Our quantitative assessment of the ROM builds the foundation for further computational investigations into the locomotor capabilities of archosaurs. While the osteological ROM is a useful tool to exclude certain possibilities in extinct species, its informational value is limited as it can only exclude osteologically impossible postures (e.g.^20,46,51^), leaving a very wide range of possibilities in the case of *Euparkeria*. The remaining ROM then can only be further narrowed down to in vivo conditions using soft tissue constraints; e.g. through ligamentous ROM simulations^53^ and biomechanical analyses of neuromuscular control of the hindlimb joints. Incorporation of such soft tissue parameters into digital simulations will further elucidate the pattern of hindlimb postural evolution in archosaurs.

## Methods

### μCT-scanning

**Figure 8:**
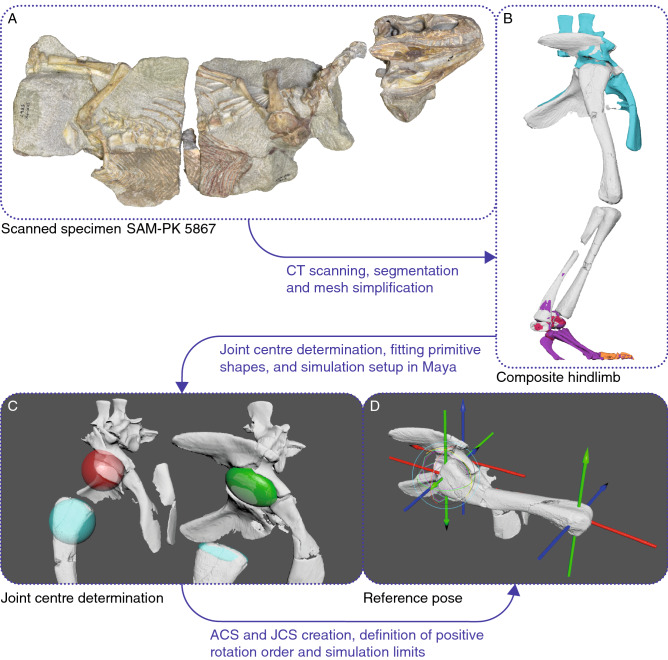
Methodological approach of this study. **(A)** Specimen selection for CT-scanning, image courtesy of R. Butler. **(B)** Composite pelvis and hindlimb based on SAM PK 5867 (white), SAM PK 5867 mirrored from opposite side (orange), SAM PK 6047A (blue), SAM PK K8309 (purple) and UMZC T.692 (red). **(C)** Geometric primitive shapes fitted to the acetabulum and femoral head to determine the joint centres. **(D)** Simulation setup in Maya in the reference pose. *ACS* anatomical coordinate system, *JCS* joint coordinate system. A detailed description of the reference pose, with the ACS and JCS setup is in the Supplementary Information.

High-resolution micro-CT scans of several specimens of *Euparkeria* were obtained from the SAM and the UMZC (Figs. 2, 8A). Specimens SAM PK 5867, SAM PK 6047A and SAM PK K8309 were μCT scanned at the Stellenbosch University, Stellenbosch, South Africa, using a General Electric VTomex L240^72^, and UMZC T.692 (also known as ‘Watson’s specimen A’ and formerly R 527)^40,73^, was μCT scanned at the University of Cambridge, Cambridge, UK, with a Nikon Metrology XT H 225 ST; see Table 1 for all scan parameters.

**Table 1:** μCT scan settings and scan resolutions for all datasets.

Specimen	Voltage (kV)	Current (µA)	Exposure duration (ms)	Voxel size (μm)	Number of slices	Resolution (pixels)
SAM PK 5867	170	400	500	90	1792	1,387 × 515
SAM PK 6047A	170	400	500	90	1618	673 × 971
SAM PK K8309	170	400	500	50	1617	1,206 × 550
UMZC T.692 1	115	120	1,000	200	1568	1,266 × 1,197
UMZC T.692 2	115	120	1,000	200	1799	1,447 × 1,301
UMZC T.692 3	190	170	1,415	200	1999	1,418 × 2000

The scanned blocks of both SAM PK 5867 and SAM PK 6047A are the ones containing the pelvis. UMZC T.692 1, 2 and 3 correspond to ankle bones scan 1 and 2 and the scan of the foot block.

### Segmentation and 3D model generation

The datasets of the scanned specimens were imported as TIFF stacks into Avizo 9.7 Lite (Thermo Fisher Scientific Inc, Waltham, USA; https://www.thermofisher.com/ch/en/home/industrial/electron-microscopy/electron-microscopy-instruments-workflow-solutions/3d-visualization-analysis-software/avizo-materials-science.html.html) for segmentation and 3D model generation. Each dataset was segmented manually by tracing the bone in the individual CT slices in a single axis and corrected, where necessary, in the perpendicular planes (see^47^), as the inbuilt automated segmentation algorithms of Avizo could not be applied due to varying X-ray attenuation within the bones themselves and noise artefacts bleeding into the density spectrum of the surrounding matrix. Minor taphonomic artefacts; e.g. cracks within the bones; were filled in by interpolation of bordering bone and/or manual correction following Lautenschlager^74^. Displaced elements due to larger breaks were segmented individually and digitally rearticulated later on. High-resolution meshes of the bones were decimated in MeshLab^75^ 2016 (https://www.meshlab.net/) using the Quadric Edge Collapse Decimation filter and cleaned from non-manifold geometry and self-intersecting faces with the cleaning and repairing filters in MeshLab before the bones were articulated in Autodesk Maya 2017 (Autodesk Inc., San Rafael, CA, USA; https://www.autodesk.com/products/maya/). We then created a nearly complete composite pelvic girdle and hindlimb by combining the skeletal elements of all scanned specimens (Fig. 8B), missing only a few distal phalanges, which could not be reconstructed as they were either partially eroded or absent from all specimens. All specimens were scaled to the most complete specimen, SAM PK 5867 (Table 2). As potentially allometric scaling factors could not be assessed due to the small sample size, an isometric growth pattern had to be assumed for *Euparkeria* (see^46^, Supplementary Information).

**Table 2:** Isometric scaling factors.

Specimen	Scaling factor	Overlapping elements
SAM PK 5867	1	–
SAM PK 6047A	0.85	Pelvic girdle
SAM PK K8309	0.92	Tibia, fibula, tarsals and metatarsals
UMZC T.692	0.76	Tarsal bones, humerus, radius, ulna

### Joint centre determination

Geometric primitive shapes—e.g. cylinders, ellipsoids, spheres or planes—were fitted to the articular surfaces (Supplementary Fig. S8) of the investigated joints to establish their centre of rotation following the methods of Bishop, Cuff & Hutchinson^76^. The faces of the articular surfaces were selected and isolated in Maya to be fitted with primitive shapes in MATLAB (The MathWorks, Inc., Natick, MA, USA). The vertices of any imported mesh acted as point clouds to which the primitive shapes were fitted. The superimposed geometric centres of two (or potentially more) resulting shapes from two articulating segments represented the centre of the respective joint. A sphere and an ellipsoid were fitted to both the acetabulum and the femoral head to test the influence of different fitted primitive shapes to the articular surfaces (Fig. 8C). The centre for the ankle/crural LAR joint was determined by fitting a sphere to the distal articular surface of the fibula. However, due to the complexity of the mesotarsal ankle joint, the distal ankle FE joint could not be determined using fitted primitive shapes. Therefore, the position of the joint centre and the rotation axis were back-calculated from animated motion around the joint (see Supplementary Information).

### Simulation setup in Maya

Ball and socket joints, e.g. hip and shoulder joints, are highly mobile and complex motion during locomotion results from interaction between all three degrees of freedom (DOFs)^52,55,59,77^. Solely linear representation of joint movement (e.g.^46,47^) is insufficient to capture the complexity of the multidimensional movement in these joints and either neglects large sections of potential ROM, only enabled through combination of multiple DOFs, or erroneously increases the ROM due to the addition of all DOFs without considering their interactions and thus leads to the inclusion of ROM ‘corners’. Therefore a 3D joint sampling approach, following the methodology of Manafzadeh & Padian^53^ was performed to estimate the 3D ROM in the hip joint of *Euparkeria*.

Based on the geometric primitive shapes (Fig. 8C), an anatomical coordinate system (ACS) for each segment and its joint surfaces, and a joint coordinate system (JCS) for each joint (computed by joining 2 ACSs), were created in Autodesk Maya, following the approach by Kambic, Roberts & Gatesy^52^ using XROMM MayaTools 2.2.3^78,79^. A forward kinematic rig was created in Maya, following Manafzadeh & Padian^53^ and A. R. Manafzadeh pers. comm. (2019). The specimen was articulated in a default reference pose, with all joint rotations set to 0° (Fig. 8D) following Kambic, Roberts & Gatesy^52^. To establish the coordinate system for correct rotation orders and to match the ‘right hand rule’ conventions for counter-clockwise positive rotation (e.g.^45,52,53^) a second joint was created with a pre-set rotation of 0°/0°/90° to which the hip joint was parented (A. R. Manafzadeh pers. comm. 2019), thus ensuring desired behaviour. To test different joint setups, based on the set of different fitted geometric primitive shapes, and to accommodate for the uncertain amount of epiphyseal cartilage (e.g.^26,80,81^), a dynamic rig was created. Using multiple dropdown menus in Maya, the specimen and the individual primitive shapes could be selected, and the percentage of additional articular cartilage, based on the femur length, could be changed through a slider, with the JCS and joint centres automatically adjusting to the selected parameters (see Supplementary Information).

For each joint setup, all possible combinations of joint rotation, ranging from − 180° to 180° for flexion/extension (FE), − 90° to 90° for abduction/adduction (ABAD) and − 180° to 180° for long-axis rotation (LAR) at 5° increments were systematically sampled. Potential mesh interpenetration, between the pelvic girdle and femur, was automatically detected using a Boolean operation in Maya and thus resulted in an inviable pose^53^. In our simulations we encountered an abnormal behaviour of the Boolean operation in Maya 2017 and newer versions. Occasionally, the Boolean operation did not automatically update each frame of the animation and got stuck unless one of the intersecting meshes received a translation input. A work-around using an additional expression to force the Boolean operation to update for each frame, to address this issue should it arise, is outlined in the Supplementary Information.

## Supplementary information

Supplementary Information 1.

Supplementary Dataset 1.

Supplementary Video 1.

Supplementary Video 2.

Supplementary Video 3.

## Data Availability

The datasets generated during the current study are included in this published article (and its Supplementary Information files). The CT datasets used in this study to generate the 3D models of *Euparkeria* are available from Figshare under the following link: 10.6084/m9.figshare.12283811.
