# Supplementary material for: 3D hindlimb joint mobility of the stem-archosaur Euparkeria capensis with implications for postural evolution within Archosauria
Source: Sci Rep. 2020 Sep 21;10:15357. doi: 10.1038/s41598-020-70175-y (PMC7506000; doi:10.1038/s41598-020-70175-y)
Supplement: Supplementary file 1 — Supplementary Information 1. [file 41598_2020_70175_MOESM1_ESM.pdf]

Supplementary Information

3D hindlimb joint mobility of the stem-archosaur *Euparkeria capensis* with implications for postural evolution within Archosauria

Oliver E. Demuth[1,2,*], Emily J. Rayfield[1] & John R. Hutchinson[2]

[1]School of Earth Sciences, University of Bristol, Wills Memorial Building, Queens Road, BS8 1RJ, UK

[2]Structure and Motion Laboratory, Department of Comparative Biomedical Sciences, The Royal Veterinary College, Hawkshead Lane, Hatfield, AL9 7TA, UK

*Correspondence: odemuth@rvc.ac.uk

CONTENTS

SUPPLEMENTARY FIGURES S1-S9 3

SUPPLEMENTARY TABLES S1-S5 12

SUPPLEMENTARY NOTES (WITH SUPPLEMENTARY FIGURES S10-S16) 16

Institutional Abbreviations 16

ACS and JCS Setup 16

Pelvic ACSs. 16

Femoral ACSs. 17

Ankle ACSs 17

Hip JCS. 19

Ankle JCSs. 19

Establishing the optimal hip joint setup 20

Influence of sensitivity setup on results 23

Flexion/Extension axes non-alignment 28

SUPPLEMENTARY METHODS (WITH SUPPLEMENTARY FIGURES S17-S18) 30

Detailed instruction for the rig setup in Autodesk Maya 30

Primitive shape selector and joint superposition. 30

Boolean specimen selector. 33

Cartilage slider. 35

Force Boolean update work-around 37

Calculation of joint spacing 38

SUPPLEMENTARY REFERENCES 39

SUPPLEMENTARY FIGURES S1-S9

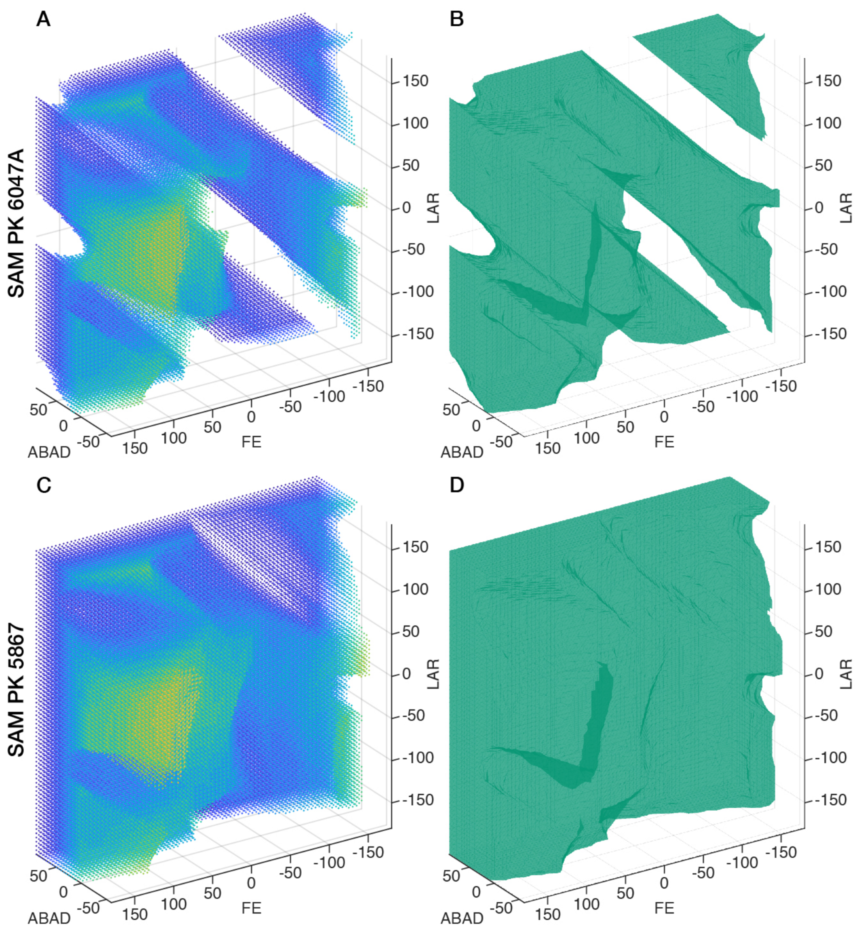

Supplementary Figure S1. ROM analysis for simulations SE0. A, C, ROM map for all viable poses. B, D, alpha shapes of the ROM maps used to calculate the volume. See Supplementary Tables S1-S2 for detailed information.

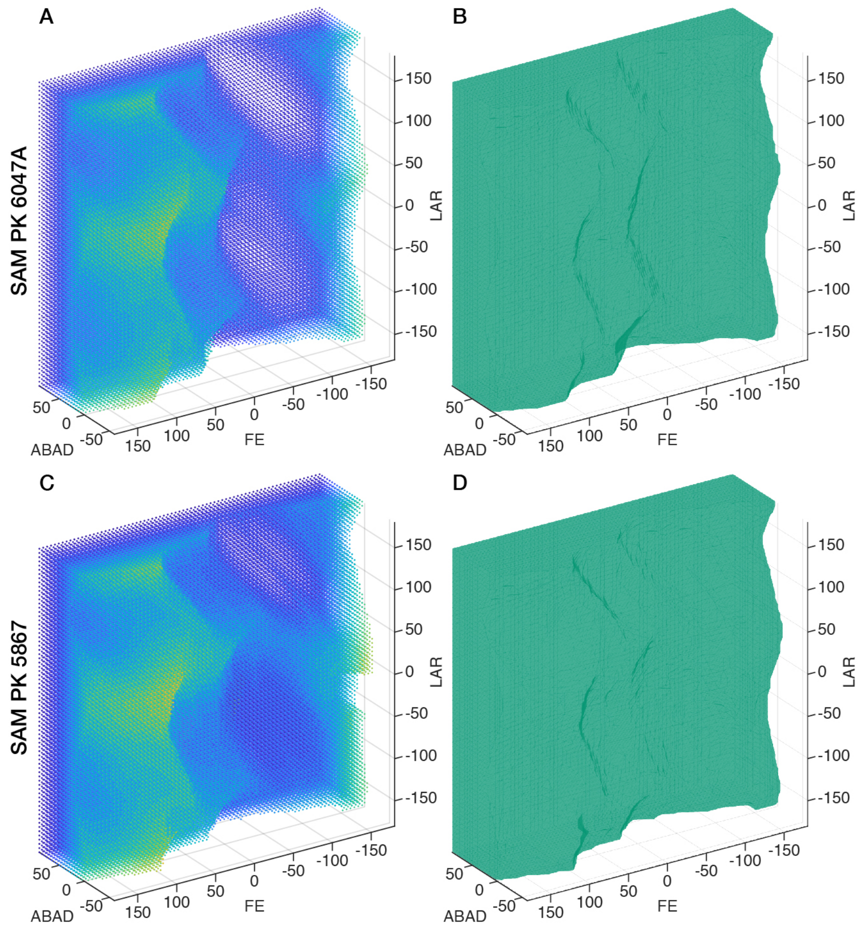

Supplementary Figure S2. ROM analysis for simulations SE10. A, C, ROM map for all viable poses. B, D, alpha shapes of the ROM maps used to calculate the volume. See Supplementary Tables S1-S2 for detailed information.

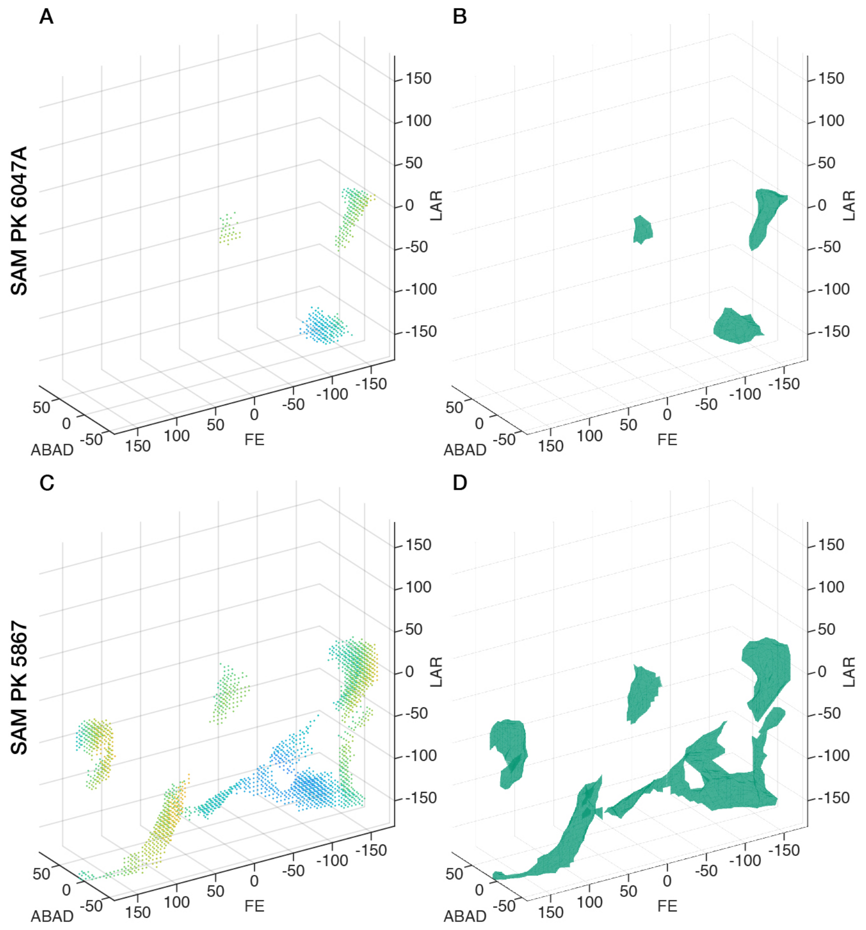

Supplementary Figure S3. ROM analysis for simulations SS0. A, C, ROM map for all viable poses. B, D, alpha shapes of the ROM maps used to calculate the volume. See Supplementary Tables S1-S2 for detailed information.

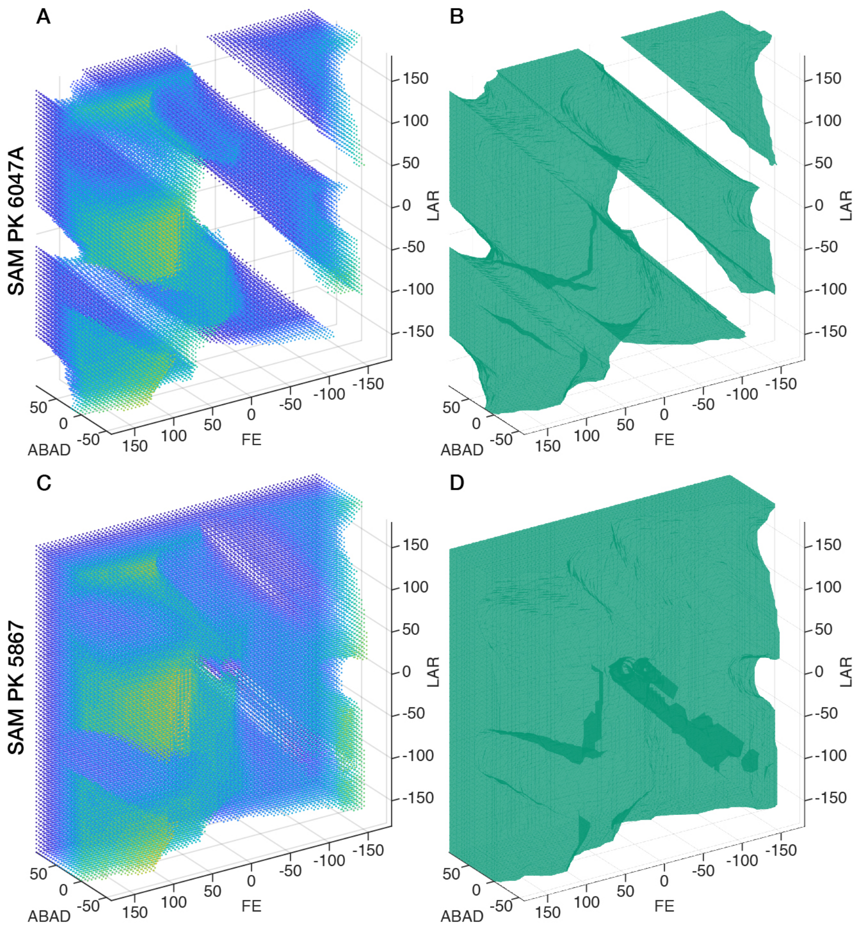

Supplementary Figure S4. ROM analysis for simulations SS10. A, C, ROM map for all viable poses. B, D, alpha shapes of the ROM maps used to calculate the volume. See Supplementary Tables S1-S2 for detailed information.

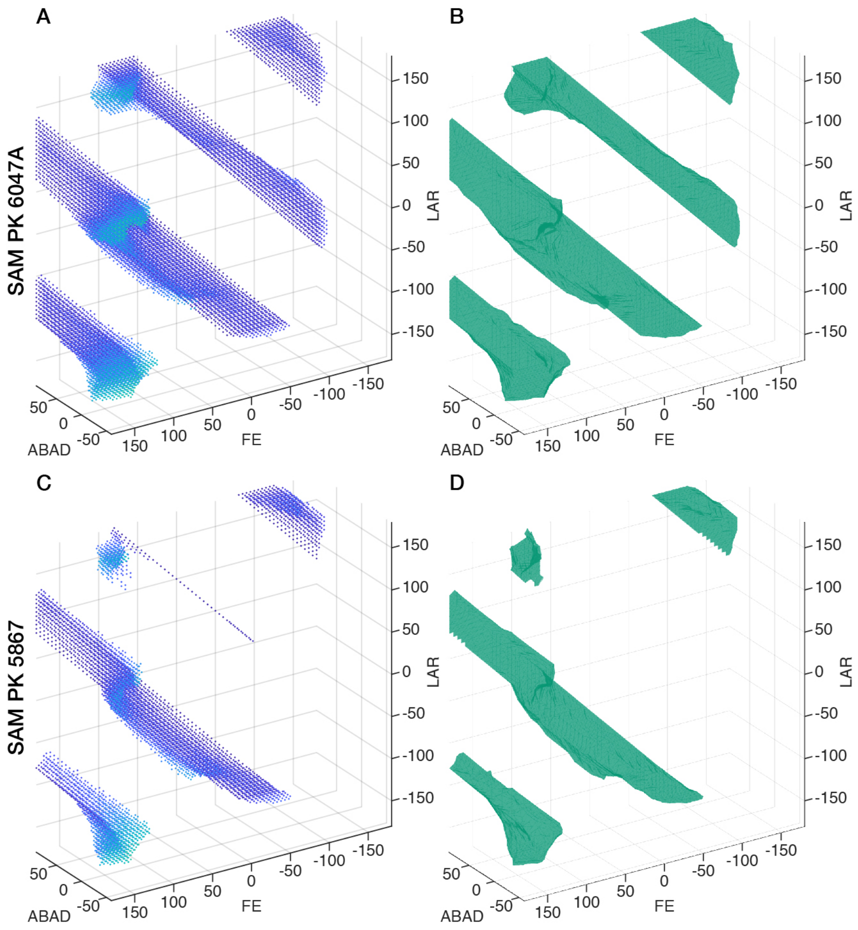

Supplementary Figure S5. ROM analysis for simulations ES10. A, C, ROM map for all viable poses. B, D, alpha shapes of the ROM maps used to calculate the volume. See Supplementary Tables S1-S2 for detailed information.

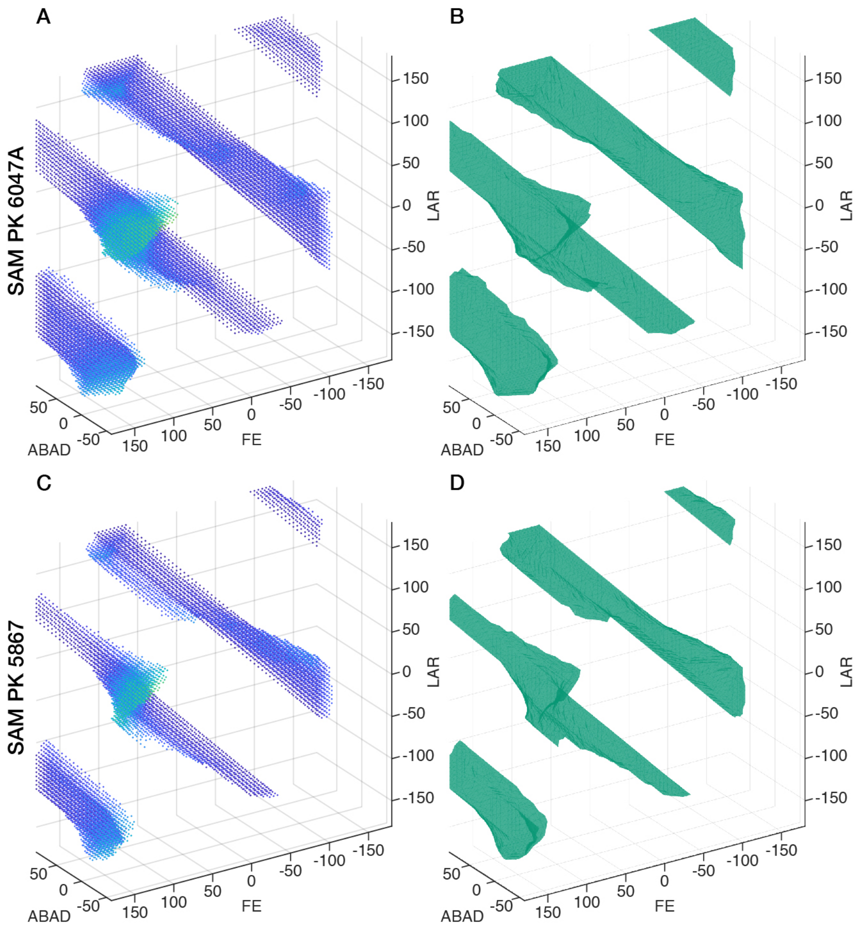

Supplementary Figure S6. ROM analysis for simulations EE0. A, C, ROM map for all viable poses. B, D, alpha shapes of the ROM maps used to calculate the volume. See Supplementary Tables S1-S2 for detailed information.

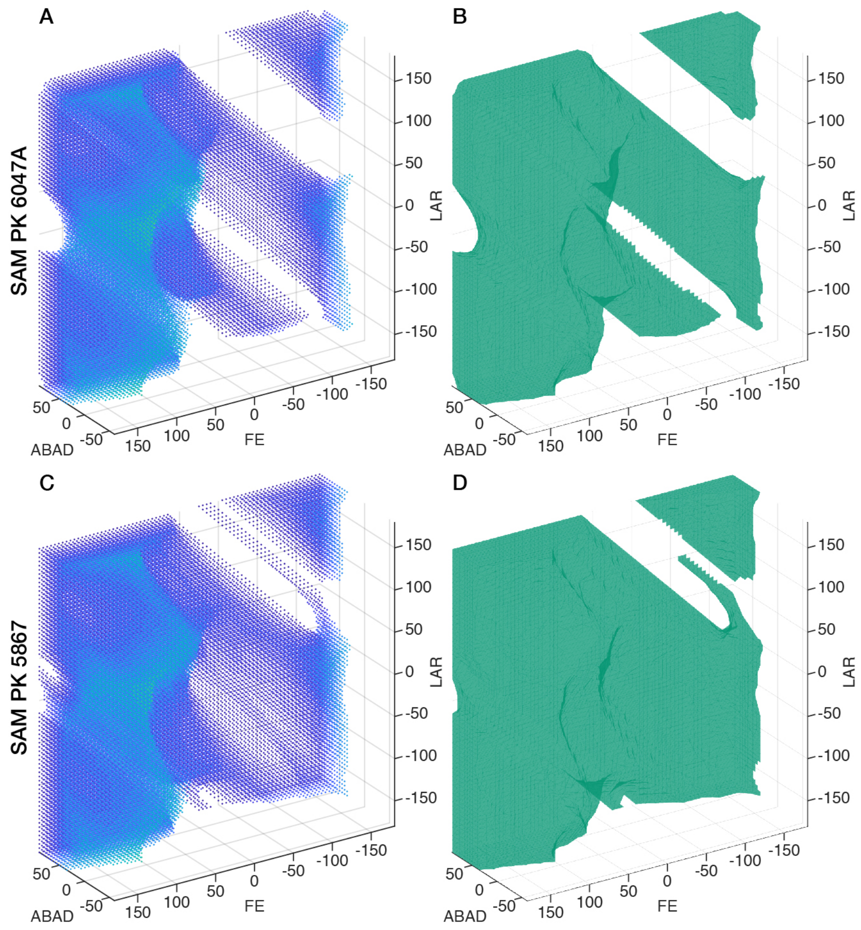

Supplementary Figure S7. ROM analysis for simulations EE10. A, C, ROM map for all viable poses. B, D, alpha shapes of the ROM maps used to calculate the volume. See Supplementary Tables S1-S2 for detailed information.

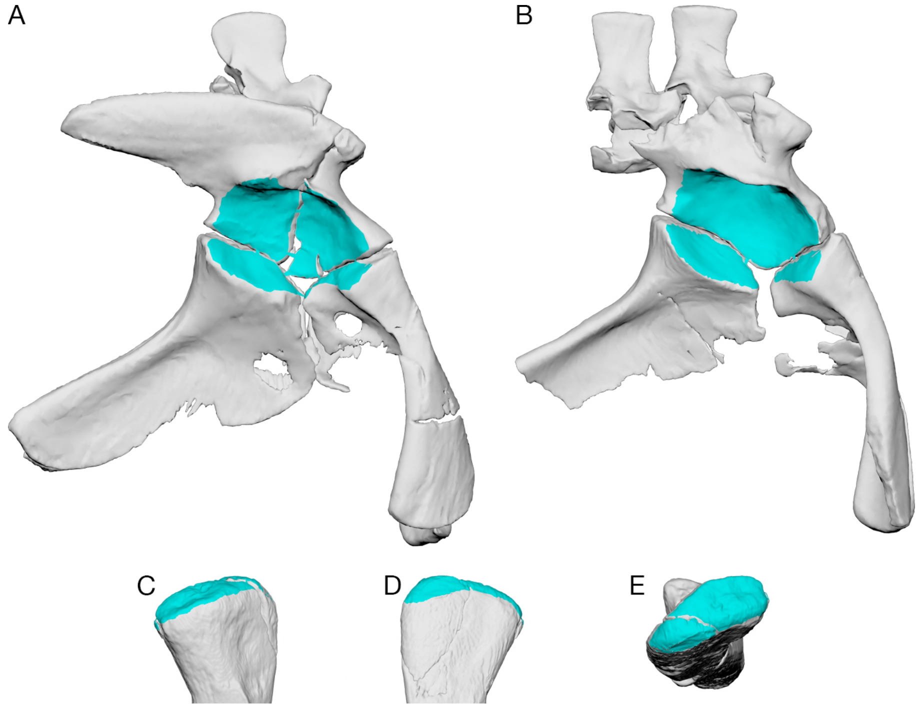

Supplementary Figure S8. Highlighted articular surfaces used for the primitive shape – fitting at the hip joint. Acetabulum articular surface of SAM PK 5867 (A) and SAM PK 6047A (B), articular surface of the femoral head in medial (C), lateral (D) and proximal view (E). Note the damaged supra-acetabular rim in SAM PK 5867.

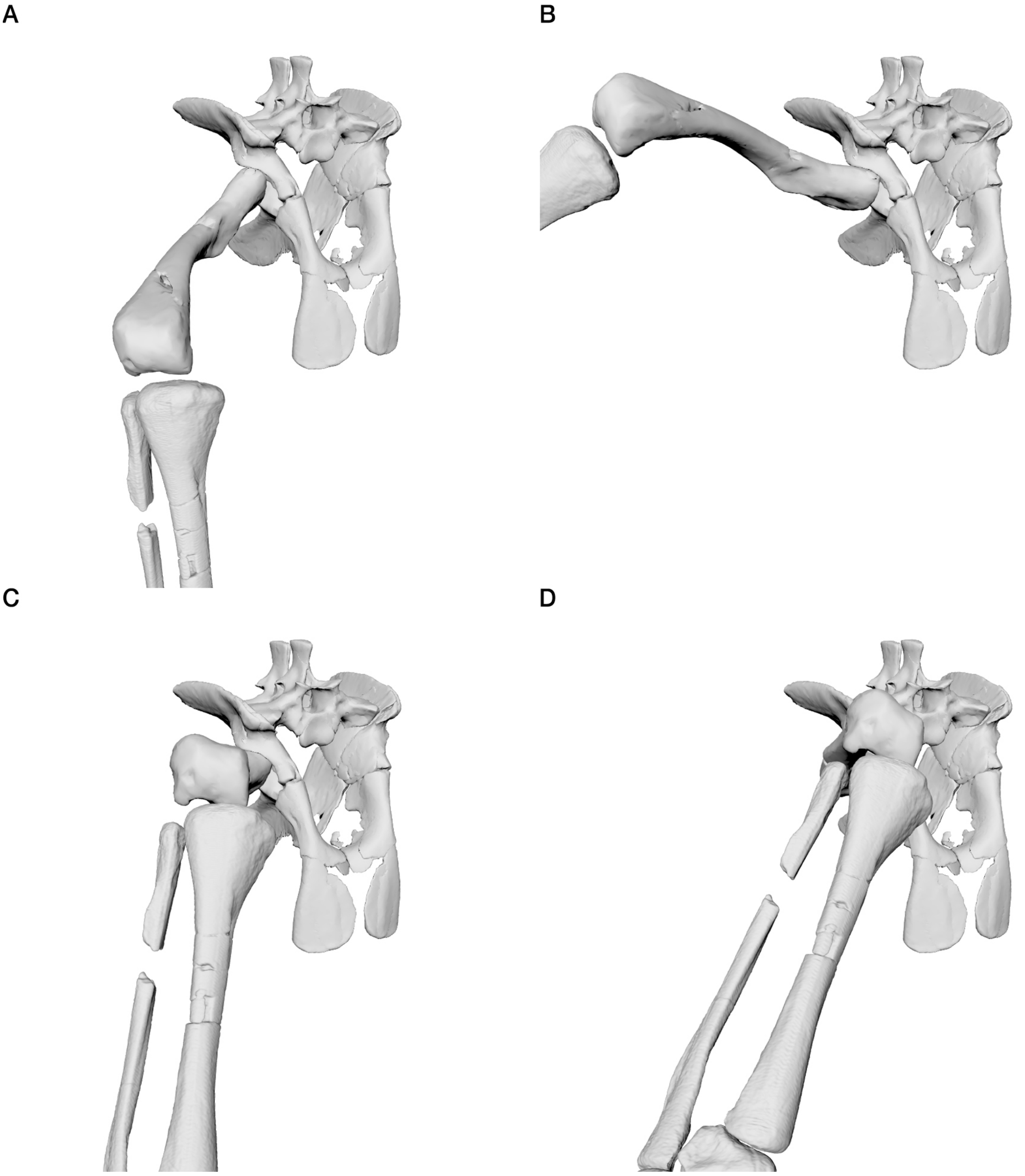

Supplementary Figure S9. Exemplary osteologically impossible sprawling poses based on the ROM analysis. The poses are described by (LAR/ABAD/FE) coordinates in degrees. (A), 10/40/15 (B), -80/60/-50 (C), -20/30/-15 (D), -40/20/-20.

SUPPLEMENTARY TABLES S1-S5

Supplementary Table S1. Results of the pelvic ROM simulation for specimen SAM PK 6047A.

Simulation	Viable poses	Volume [degrees^3]	Alpha radius [degree]
**SE0**	58776	6.67E+06	5√3
**SE10**	71819	8.20E+06	5√3
**SS0**	365	3.02E+04	5√3
**SS10**	57090	6.45E+06	5√3
**ES0***	NA	NA	NA
**ES10**	12111	1.22E+06	5√3
**EE0**	14501	1.49E+06	5√3
**EE10**	39477	4.36E+06	5√3

*Simulation ES0 was not performed, as no viable poses could have been generated due to inevitable constant collision of the femur with the pelvis. The alpha radius was set as the diagonal length of a cube with a side length of the degree interval specified for the simulation in Maya.

Supplementary Table S2. Results of the pelvic ROM simulation for specimen SAM PK 5867.

Simulation	Viable poses	Volume [degrees^3]	Alpha radius [degree]
**SE0**	81079	9.35E+06	5√3
**SE10**	79971	9.21E+06	5√3
**SS0**	2159	1.81E+05	5√3
**SS10**	80518	9.32E+06	5√3
**ES0***	NA	NA	NA
**ES10**	5631	5.37E+05	5√3
**EE0**	9029	9.03E+05	5√3
**EE10**	42021	4.64E+06	5√3

*Simulation ES0 was not performed, as no viable poses could have been generated due to inevitable constant collision of the femur with the pelvis. The alpha radius was set as the

diagonal length of a cube with a side length of the degree interval specified for the simulation in Maya.

Supplementary Table S3. Fitted primitive shape radii for *Crocodylus niloticus* and *Euparkeria capensis*.

Specimen	Femoral ellipsoid average radius [mm]	Femoral sphere radius [mm]	Acetabulum elipsoid average radius [mm]	Acetabulum sphere radius [mm]
Crocodylus niloticus				
DDNC04	6.444	8.337	7.321	9.996
DDNC06	6.236	6.731	7.962	9.587
DDNC09	6.457	13.080	9.442	10.035
DDNC10	5.907	8.758	9.325	11.114
Euparkeria capensis				
SAM PK 5867	4.197	7.602	5.997	7.799
SAM PK 6047A*	4.197	7.602	6.140	7.446

*SAM PK 6047A was isometrically scaled to match SAM PK 5867. SAM PK 6047A has no preserved femur; therefore the femur of SAM PK 5867 was used.

Supplementary Table S4. Joint spacing for different joint setups.

Specimen	Joint setup	Centroid distance [mm]	Joint spacing [mm]	Superimposed joint spacing [mm]
Crocodylus niloticus				
DDNC04	EE	7.310	8.187	0.877
	ES	9.920	8.904	-1.016
	SE	1.402	4.954	3.552
	SS	4.277	5.935	1.659
DDNC06	EE	8.268	9.993	1.726
	ES	9.113	10.344	1.231
	SE	2.067	5.418	3.351
	SS	2.874	5.730	2.856
DDNC09	EE	5.387	7.863	2.985
	ES	8.663	8.198	-0.465
	SE	1.862	5.737	3.875
	SS	4.088	5.022	0.934
DDNC10	EE	3.526	6.944	3.418
	ES	7.790	8.357	0.567
	SE	1.811	7.018	5.207
	SS	4.164	6.521	2.357
Euparkeria capensis				
SAM PK 5867	EE0	0	1.800	1.800
	EE10	4.884	6.684	1.800
	ES0	0	-1.605	-1.605
	ES10	4.884	3.279	-1.605
	SE0	0	3.602	3.602
	SE10	4.884	8.486	3.602
	SS	0	0.197	0.197
	SS10	4.884	5.081	0.197
SAM PK 6047A*	EE0	0	1.943	1.943
	EE10	4.884	6.827	1.943
	ES0	0	-1.462	-1.462
	ES10	4.884	3.422	-1.462
	SE0	0	3.249	3.249
	SE10	4.884	8.134	3.249
	SS0	0	-0.156	-0.156
	SS10	4.884	4.729	-0.156

*SAM PK 6047A was isometrically scaled to match SAM PK 5867. The joint setup is named after the primitive shapes fitted to the acetabulum and femoral head. First the fitted shape to the acetabulum is indicated and second the fitted shape to the femoral head. For example in the joint setup SE a sphere (S) was fitted to the acetabulum and an ellipsoid (E) to the femoral

head. In *Euparkeria* the additional cartilage is also indicated, as either 0% (0) or 10% of femoral length (10).

Supplementary Table S5. Flexion/Extension axes offsets

Joint	local FE [degrees]	local ABAD [degrees]	local LAR [degrees]	FE axis local X-axis offset [degrees]	FE axis local Y-axis offset [degrees]	FE axis global X-axis offset [degrees]	FE axis global Y-axis offset [degrees]
Hip joint	77	25	-6	0.0	0.0	0.0	0.0
Knee joint	-36	0	0	0.0	-7.9	1.8	18.1
Distal ankle (mesotarsal) joint	65	0	0	20.2	28.2	-29.1	2.2
Metatarsophalangeal joint	-38	0	0	-7.8	10.5	-16	1.7

Angles were computed for local offset (in relation to the parent element) and global offset (in relation to the world origin) for the most adducted posture possible of *Euparkeria*.

SUPPLEMENTARY NOTES (WITH SUPPLEMENTARY FIGURES S10-S16)

Institutional Abbreviations

IFGT, Institute for Geosciences, Eberhard-Karls-Universität Tübingen, Tübingen, Germany (formerly Geologisch-Paläontologisches Institut Tübingen: **GPIT**); **NHMUK**, Natural History Museum, London, UK; **NMT**, National Museum of Tanzania, Dar es Salaam, Tanzania; **SAM**, Iziko South African Museum, Cape Town, South Africa; **UMZC**, University Museum of Zoology Cambridge, Cambridge, UK.

ACS and JCS Setup

The ACS and JCS were created following the conventions of Kambic, Roberts & Gatesy[1]. The location of the ACS was determined by the centroid of the primitive shapes fitted to the articular surfaces of the pelvis and femur. The ankle ACSs and JCSs were computed differently.

Pelvic ACSs.

The pelvic ACS was created in the centre of the pelvis, midway between both acetabula. The positive z-axis was dorsally directed and the *X-axis* caudally, thus the *Y-axis* pointed towards the right acetabulum. The hip ACS was created in the acetabular centroid and determined by the fitted shapes, thus its position varies depending on weather the sphere or ellipsoid was the active primitive shape. The *Z-axis* pointed medially through the acetabulum and X-axis anteriorly, which resulted in the *Y-axis* pointing ventrally in the right-handed coordinate system.

Femoral ACSs.

For the femur, two ACS were created proximally in the femoral head and distally in the condyle (Supplementary Fig. S10). While the proximal ACS determined the long-axis, the positive *X-axis* passed through the centre of the distal and proximal fitted primitive shapes. The distal ACS determined the natural direction regarding long-axis rotation, as the positive *Z-axis* passed through the centres of both distal condyles, as if they lay flat on a surface. Thus the *XZ-planes* of both ACS corresponded and the positive *Y-axis* points dorsally in the proximal ACS and ventrally in the distal ACS. As the proximal ACS is positioned in the centre of the active primitive shape, its position changes relative to the femur, depending on which primitive shape is selected (Supplementary Fig. S10B, C).

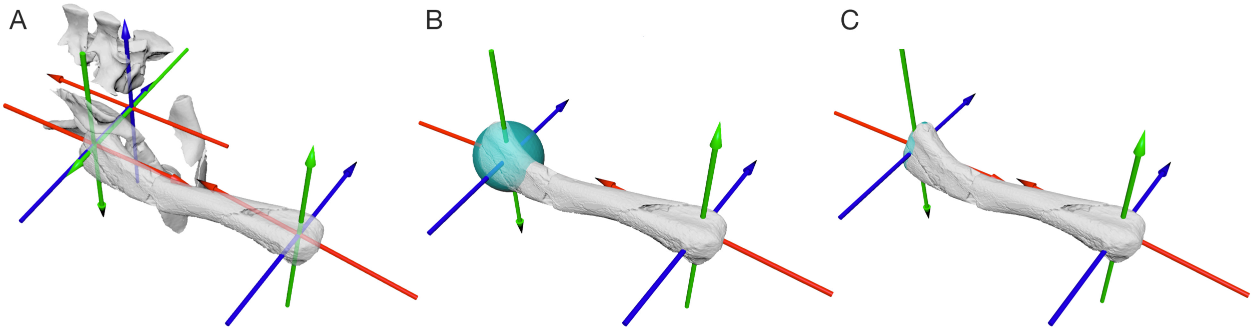

Supplementary Figure S10. ACS and JCS setup. A, ACS configuration and reference pose (all rotations zeroed) of the pelvis. B, ACSs of the femur with the sphere fitted to the femoral head. C, ACSs with the ellipsoid fitted to the proximal femur. Note the influence of the active primitive shape on the position of the proximal ACS. *X-axis*, red; *Y-axis*, green; *Z-axis*, blue.

Ankle ACSs

The ankle ACS consisted of two parts, the proximal ACS in the distal fibula (LAR) and the distal ACS in the mesotarsal ankle joint (FE). The two rotation axes did not meet in the 3D

space and therefore needed to be modelled as two separate joints. The astragalus was assumed fixed to the tibia while the fibula was able to rotate around the astragalus and calcaneum. Therefore LAR of the crus resulted in motion of the "tibiotarsus" around the fibula. The proximal ACS was placed in the centroid of the sphere fitted to the distal articular surface of the fibula. To derive the LAR joint axis, the (negative) *X-axis* was aimed at the medial femoral condyle, which guaranteed that the contact between the tibia and femur was maintained during motion (Supplementary Video S2).

The distal ankle ACS could not be determined using fitted primitive shapes due to the complex morphology of the mesotarsal joint and the numerous bones contributing to it. Therefore, the position and orientation of the ACS was reverse-calculated from animated motion around the joint. The distal tarsals and metatarsals were translated and rotated around the ankle joint from a fully flexed to a fully extended position, while avoiding disarticulation and bone collision. At 15 regular intervals the position of several points on the articular surface of the metatarsals and distal tarsals were noted. These 3D coordinates were used to fit curves along which these points followed from flexion to extension. The curvature centre of these curves was then calculated in Maya using the *pointOnCurveInfo* at 5 regular intervals between 30% and 70% of the curve, which resulted in 5 curvature centres for each of the 10 curves. A principal component analysis (PCA) was performed based on the 3D coordinates of the established curvature centres to approximate the direction of the ankle joint FE axis (Supplementary Fig. S11A). The main axis of variability (PC1), which accounted for 90.3% of the observed variability, was used as the flexion-extension axis of the ankle joint. The average position of the curvature centres was established as the distal ankle joint centre and location of the ACS. The loadings of the PC1 were used as vector in which direction the positive *Z-axis* was aimed (Supplementary Fig. S11B).

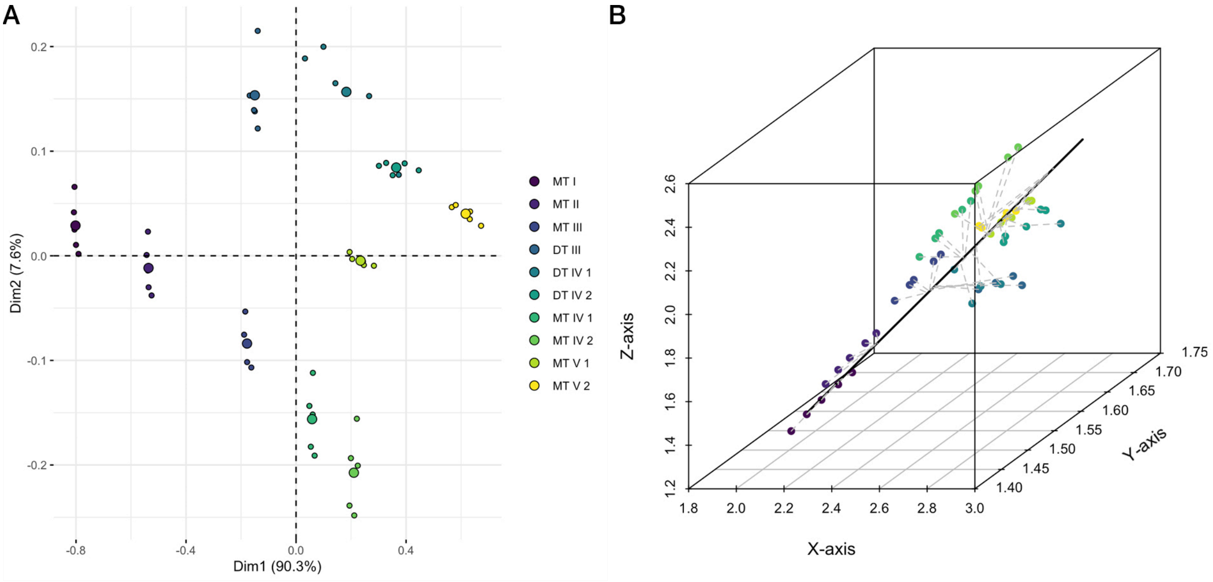

Supplementary Figure S11. Distal ankle ACS estimation. A, Principal component analysis (PCA) axes 1 and 2 for the axis estimation; B, 3D coordinates of the curvature centres for each curve and the estimated FE axis of the distal ankle joint. Abbreviations: MT, metatarsal; DT, distal tarsal.

Hip JCS.

The hip JCS was defined by the axes of the ACS in the acetabulum and the femoral head. FE resulted from rotation around the *Z-axis* of the acetabular ACS, while extension was positive and flexion negative. ABAD measured the rotation around the *Y-axis* of the proximal ACS, abduction being positive and adduction negative. LAR measured the rotation of the femur around the *X-axis* of the proximal ACS, external rotation being positive.

Ankle JCSs.

The proximal ankle JCS was based on the distal fibula ACS and allowed rotation only around the *X-axis* to measure crural LAR, the other rotation axes were locked. A right-hand rule and positive rotation for pronation and negative rotation for supination were ensured as the

negative *X-axis* was aimed proximally. Due to the setup of the joint axis, supination of the ankle joint resulted in flexion of the tibia around the knee joint, while the fibula remained 'fixed' and was only influenced by knee FE (Supplementary Video S2).

The distal ankle JCS was based on the distal ankle ACS. Ankle FE measured rotation around the *Z-axis*, all other rotation axes were locked, whereas flexion was positive and extension negative.

Establishing the optimal hip joint setup

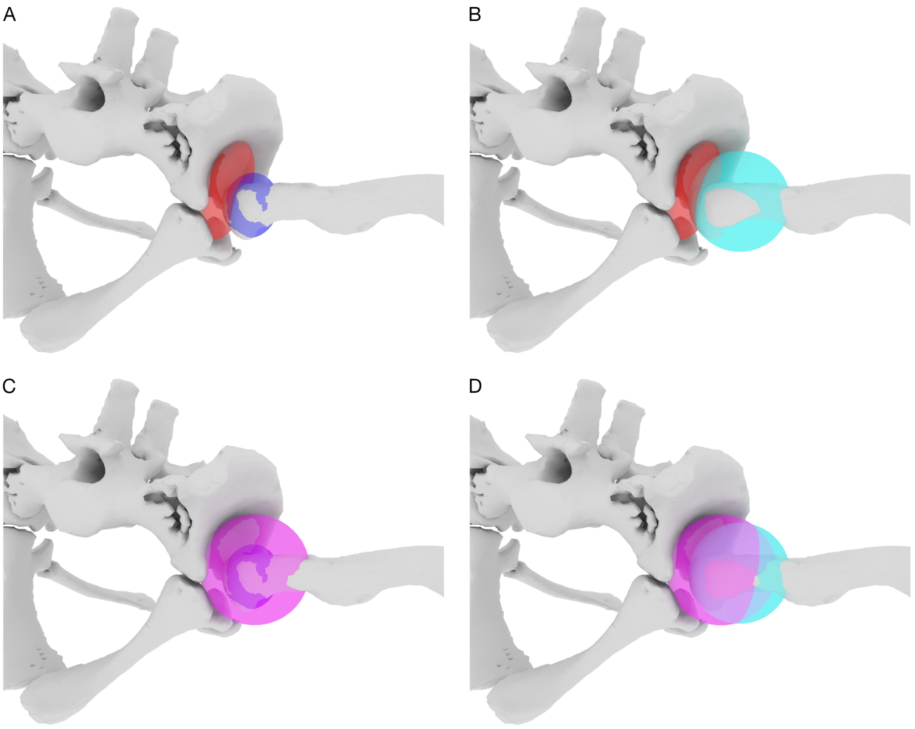

Supplementary Figure S12. Fitting of primitive shapes to an articulated crocodile hip. Fitted ellipsoids (A, B; red) and spheres (C, D; magenta) to the right acetabulum of DDNC09 in posterolateral view. Ellipsoids (C, D; blue) and spheres (B, D; cyan) fitted to the femoral head.

While the function of the hip joint as a ball and socket joint is undisputed, an interpretation of the femoral head as a sphere might not accurately predict the position of the joint centre and other primitive shapes may better capture the shape of the proximal femur - e.g. an ellipsoid - especially in the case of a lateromedially expanded femoral head as in *Euparkeria* or crocodilians[2]. We therefore tested several joint setups in the hip of four specimens of *Crocodylus niloticus* - derived from full body CT scans - to determine the most likely joint setup for *Euparkeria*. Crocodiles and *Euparkeria* show similarities in the morphology of the femur[3] and the lateromedially expanded femoral head in *Euparkeria* is analogous to the femoral head in crocodiles. Therefore the articulation of the femoral head (i.e. proximal femur; but see Tsai et al.[2]) with the acetabulum is comparable in both taxa. Spheres and ellipsoids were fitted to the articular surfaces of the femoral head and the acetabulum of the crocodiles in the *in vivo* articulation of the hip joint (Supplementary Fig. S12).

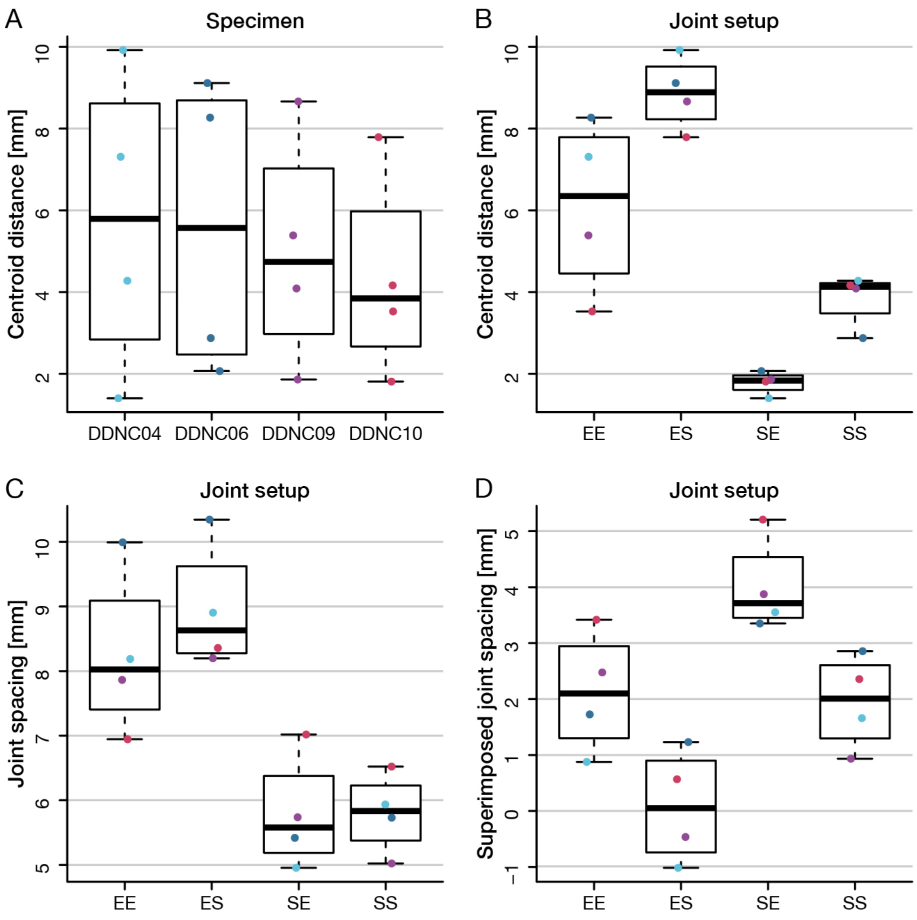

Supplementary Figure S13. Determination of best-fitting joint setup based on the four juvenile specimens of *Crocodylus niloticus*. A, distribution of the centroid distance for the individual specimens; B, centroid distance based on the joint setup; C, influence of joint setup on joint spacing; D, joint spacing for superimposed primitive shapes.

We cross-compared the distances between the centroids of the fitted spheres and ellipsoids to determine which joint setup showed the smallest distance between the centroids and is therefore closest to the actual joint centre (Supplementary Table S4; Supplementary Fig. S13). In all four specimens of *C. niloticus*, the joint setup with a fitted sphere to the acetabulum and a fitted ellipsoid to the femoral head (joint setup SE) showed the smallest distance between the centroids (Supplementary Table S4; Supplementary Fig. S13) and most accurately predicted the position of the hip joint centre. Therefore we also deemed this setup as the most

likely in *Euparkeria* and it should be preferred for all ROM simulations of archosauromorphs with a similar femoral head.

Influence of sensitivity setup on results

While we deemed simulation SAM PK 6047A SE0 as the most likely, we wanted to test how the different simulation parameters influenced the resulting ROM. Besides the different primitive shapes we decided to implement an option for additional epiphyseal cartilage due to the wide range of articular cartilage thickness in extant archosaurs[4,5] and resulting uncertainties in extinct taxa. Therefore the epiphyseal cartilage thickness was simulated in two extremes, a minimal setup derived from the primitive shapes alone with no additional cartilage and a maximum amount of additional cartilage of 10% femoral length, based on published estimates for fossil taxa[5,6], positioning the femur distally from the ACS (Supplementary Fig. S14).

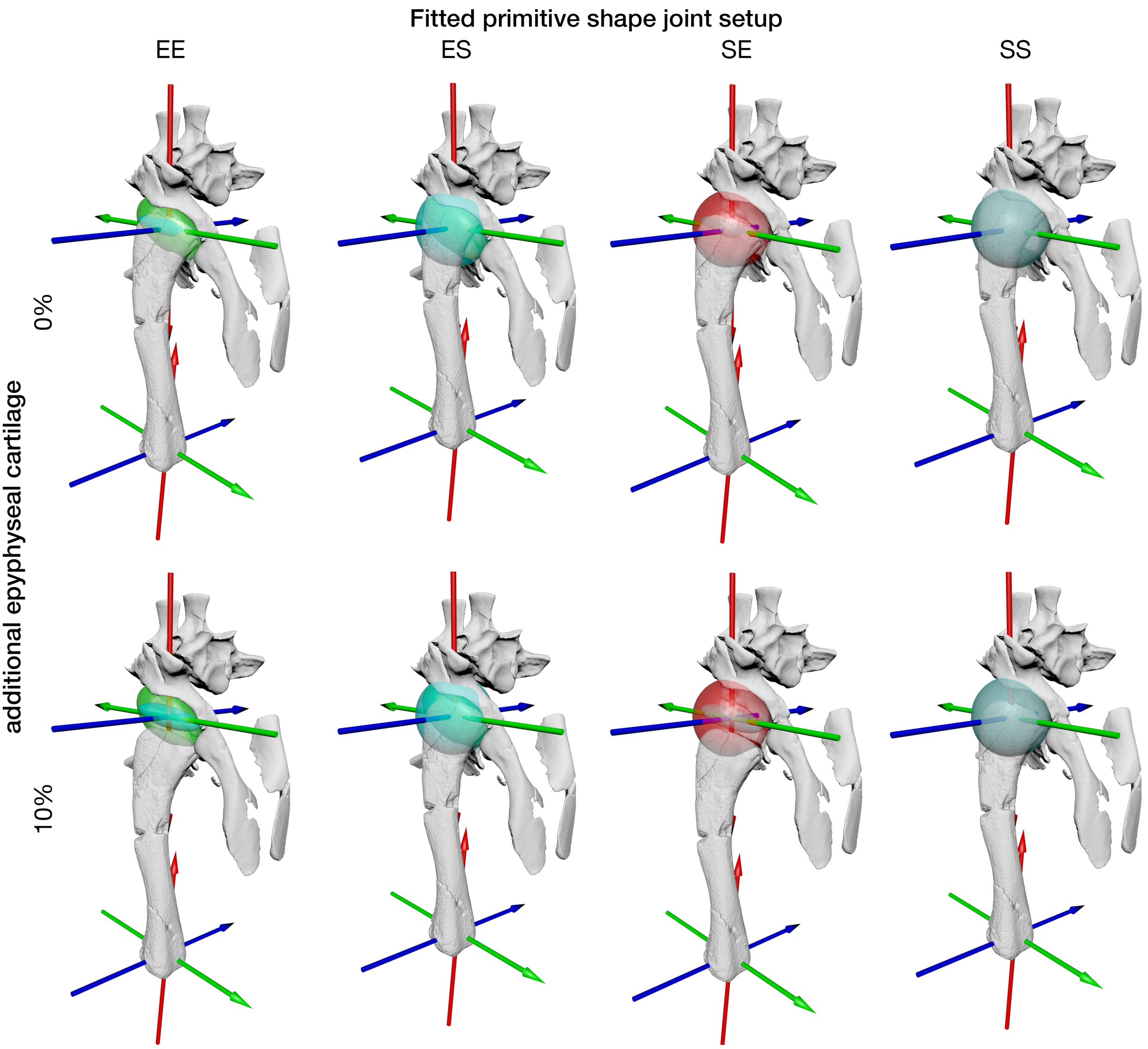

Supplementary Figure S14. Simulation setups for the sensitivity analysis of specimen SAM PK 6047A in anterolateral view. Note the large distance between the femoral head and the acetabulum in setup SE10.

While 16 simulations, depending on the specimen, fitted shapes to the acetabulum and femoral head, and additional cartilage, were set up, only 14 simulations were ultimately performed (Supplementary Figs. S1-S7). The strongly negative joint spacing (< -1 mm) in two simulations (simulations ES0) could not have resulted in any viable poses

(Supplementary Tables S1-S2) due to constant collision of the femoral head with the acetabulum.

The total number of viable poses per simulation ranged from 365 to 81,079 with the volume of the corresponding alpha shapes ranging from as low as 30,188 degrees^3 to as high as 9,353,700 degrees^3 (Supplementary Tables S1-S2). A spherical interpretation of the expanded femoral head in *Euparkeria* resulted in excessive interaction of the femoral head with the acetabulum. However, increasing the amount of simulated epiphyseal cartilage counteracted mesh interpenetration in these simulations, which brought them more in line with the joint centre determined by the fitted ellipsoid to the femoral head. The overall performance and topology of the ROM map of the spherical femoral head with additional cartilage (SS10; Supplementary Fig. S4) was similar to the elliptical femoral head without additional cartilage (SE0; Supplementary Fig. S1) in both specimens. The main differences lay in the slightly more constrained abduction/adduction (ABAD) area for moderate long-axis rotation (LAR) in SE0, whilst the ABAD values were more expanded towards more extreme values of LAR. Overall, ROM SE0 was larger than SS10 in both specimens, based on the alpha volume (0.38% for SAM PK 5867 and 3.38% for SAM PK 6047A) and the number of total viable poses (0.70% for SAM PK 5867 and 2.95% for SAM PK 6047A).

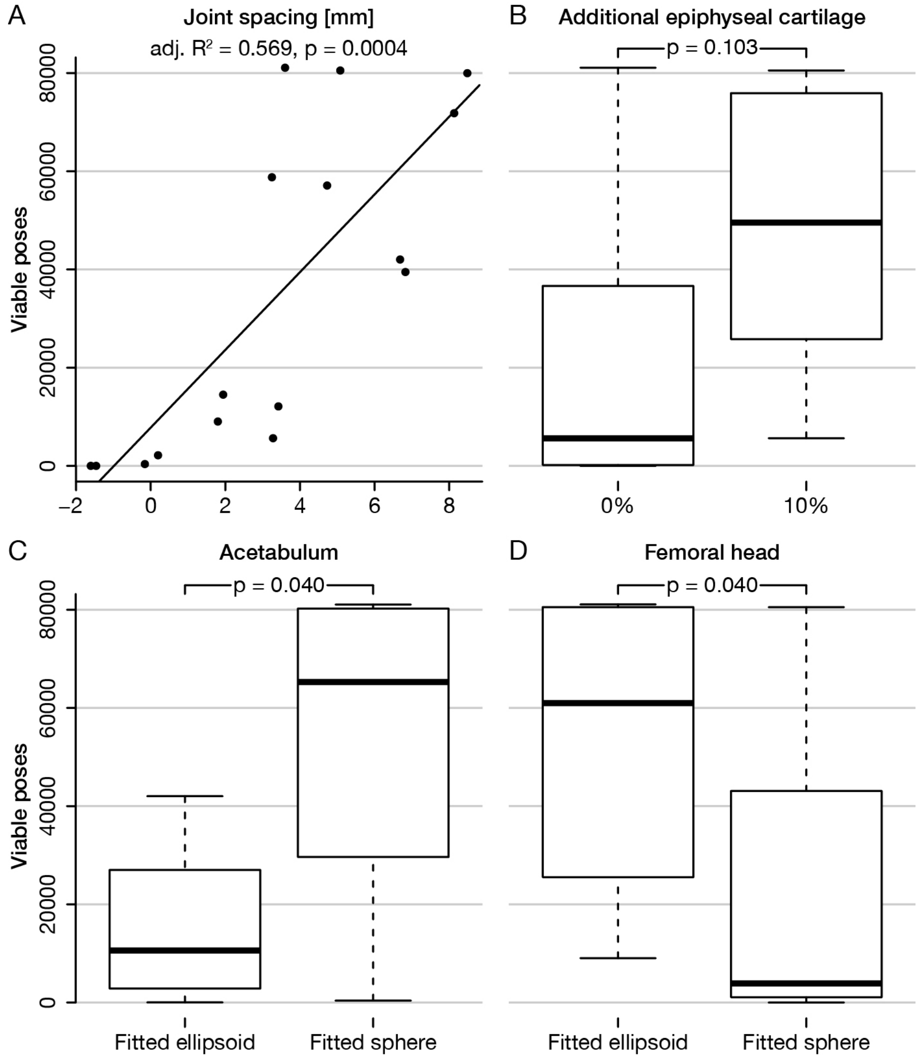

Supplementary Figure S15. Sensitivity of the viable ROM poses to the different simulation parameters. A, linear regression of viable poses against joint spacing; B, influence of additional epiphyseal cartilage on the ROM; Sensitivity of the viable poses in relation to the different fitted primitive shapes on: the acetabulum (C) and femoral head (D).

Surprisingly, a larger amount of epiphyseal cartilage did not increase the overall ROM in all simulations. The number of viable poses decreased by 1.37% from SE0 to SE10 for SAM PK 5867 as an additional 10% in cartilage was added to the femoral head (Supplementary Table S2), positioning the femur further away from the joint centre. The observed decrease resulted from the collision of the medially expanded femoral head with the supra-acetabular rim in

poses with large amounts of abduction and moderate LAR which thus became inviable, while the femoral head remained within the acetabulum when the amount of cartilage was reduced (see Supplementary Figs. S1-S2). However, due to the large joint spacing in the SE10 simulations (Supplementary Tables S1-S2), the femur appeared to have partially disarticulated from the acetabulum (Supplementary Fig. S14) and we thus deemed these simulations unlikely. In all other analyses an increase in epiphyseal cartilage resulted in a larger number of viable poses and a higher ROM volume (Supplementary Tables S1-S2), however the difference between these two subsets was not significant for the whole dataset ($p = 0.103$; Supplementary Fig. S15) or both specimens individually ($p = 0.471$ for SAM PK 5867; $p = 0.312$ for SAM PK 6047A; Supplementary Fig. S16). Simulations with a fitted ellipsoid to the acetabulum resulted in superficially substantially lower numbers of viable poses and smaller volumes than those simulations with a fitted sphere, however the difference was only slightly significant for the whole dataset ($p = 0.04$; Supplementary Fig. S15) and insignificant if both specimens were observed individually ($p = 0.194$; Supplementary Fig. S16). The same effect can be observed for the difference between the simulations with a fitted ellipsoid or sphere to the femoral head ($p = 0.04$ for both specimens; Supplementary Fig. S15; $p = 0.194$ for SAM PK 5867; $p = 0.112$ for SAM PK 6047A; Supplementary Fig. S16). This significance or insignificance was presumably an artefact of small sample size ($n = 16$ for both specimens; $n = 8$ for each specimen individually) and therefore should be taken cautiously. However, there was a strongly significant trend for a higher number of viable poses with increased joint spacing ($p = 0.0004$; Supplementary Fig. S15) even though there was a considerable amount of scatter in the data (adj. $R^2 = 0.569$).

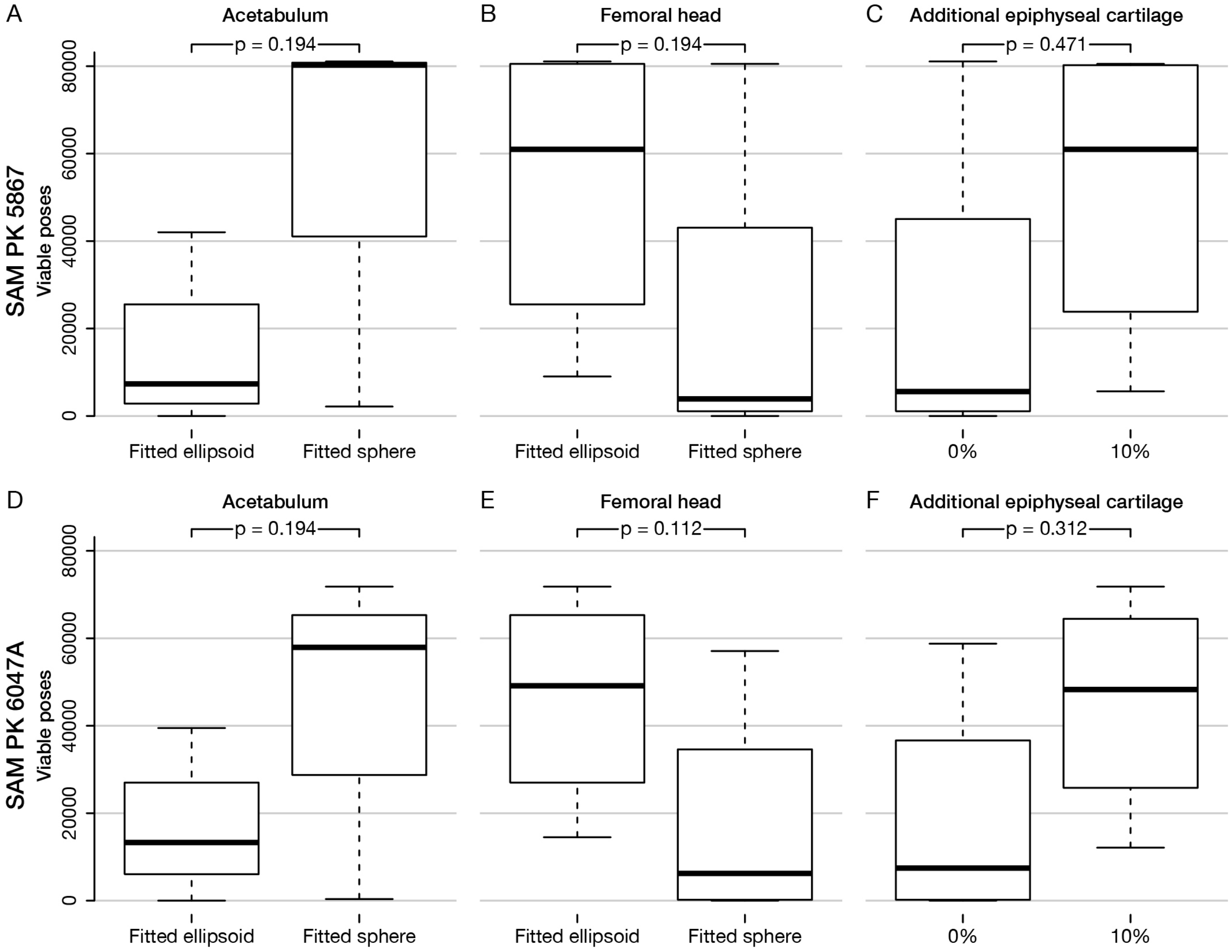

Supplementary Figure S16. Influence of the different simulation parameters on the joint poses for both specimens individually. Sensitivity of the viable poses in relation to the different fitted primitive shapes on the acetabulum (A, D) and femoral head (B, E); influence of additional epiphyseal cartilage on the ROM (C, F).

Flexion/Extension axes non-alignment

The bones were linked in Maya to form a hierarchical chain based on the ACSs (see [7,8]). The hierarchical order of the Maya rig was: pelvis > femur > fibula > tibia + proximal tarsals > distal tarsals and metatarsals with the following degrees of freedom FE/ABAD/LAR > FE > LAR > FE. In order to accomplish a parasagittal gait, all FE axes need to be aligned. In the

most adducted posture theoretically possible for *Euparkeria* (Fig. 6), these axes are not aligned (Supplementary Table S5), therefore making a parasagittal gait impossible.

SUPPLEMENTARY METHODS (WITH SUPPLEMENTARY FIGURES S17-S18)

Detailed instruction for the rig setup in Autodesk Maya

Primitive shape selector and joint superposition.

The following paragraphs guide the reader through the joint setup in Maya and explains how the dropdown menus in the *Channel Box* are created to select to automatically adjust the joint centres based on the fitted primitive shapes.

The primitive shapes imported from the Shape_Fitter MATLAB script[9] automatically correspond to the articular surfaces of the individual bones from which they have been derived. However, the pivot of the primitive shape is positioned in the world origin (0/0/0) instead of their centre. To be able to correctly superimpose the primitive shapes, e.g. of the femur and acetabulum, the pivot has to be centred first. Therefore, select the primitive shape and click on *Modify* > *Centre Pivot*, which centres the pivot in the middle of the primitive shape, which will then act as joint centre. Repeat this step for all primitive shapes within the scene.

Before the joint centres can be superimposed, the individual bones have to be parented to the respective primitive shapes, in the case of the femoral head, to both the sphere and the ellipsoid. However, make sure that all transformations on the bones are frozen (everything set to 0), under *Modify* > *Freeze Transformations*, before parenting.

If this is the case, select the first primitive shape and then the bone, e.g. femur, and in the dropdown menu (upper left corner) select *Rigging* to bring up the *Constrain* menu. Click on the box behind *Constrain* > *Parent* and the *Parent Constraint Options* windows opens. Check the *Maintain offset* and click apply. If everything worked out the bones should not have moved and the transformation attributes should have turned blue. If you now move the

primitive shape around, the bone follows. Repeat this step and parent constrain the femur to the second primitive shape.

The femur now has two constraints in the *Channel Box* as the last two entries beneath the *SHAPES* tab, both indicated with 1. This means that the femur is currently equally influenced by the joint centres of both primitive shapes. To address this issue, we can now define a dropdown menu in which the primitive shapes can be selected. Therefore, create a *Locator*, place it somewhere above the joints and rename it to *Master*. The exact position is not important but it should be easily visible, as all settings of the rig will be controlled through it. It is not necessary, but I recommend hiding all attributes before adding the new ones. With the *Master* selected click on *Modify > Add attributes*. Thus a window opens which is called "Add Attributes: |Master". Name it *FemoralHeadShape*, choose *Enum* in the *Data Type* and rename *blue* and *green* in the *Enum Names* into *Ellipsoid* and *Sphere* and click Ok. Now, a dropdown menu called "Femoral Head Shape" has appeared in the *Channel Box* of the *Master*. Even though "Ellipsoid" or "Sphere" are displayed, Maya interprets these as 0 and 1, the first enum representing 0.

Select the *Master*, femur and both primitive shapes and open the *Node Editor*. If they don't show up automatically in the *Node Editor* load the input and output connections. To create a condition node, press tab, type *condition* and hit enter. To keep everything tidy rename it to *FemoralHeadPrimitiveCond* and set the *Operation* to *Equal*, the *Color if True* to 0, 1, 0 and the *Color if False* to 1, 0, 0. Now, through click and drag, connect the *Femoral Head Shape* attribute of the *Master* to the *First Term* attribute of the *FemoralHeadPrimitiveCond* node. On the other side connect *Out Color R* output with the second input, corresponding to the sphere on the parent constraint and the *Out Color G* with the first input. You can also connect *Out Color R* with the *Visibility* of the sphere and the *Out Color G* with the *Visibility* of the ellipsoid (Supplementary Fig. S17). Thus when the sphere is selected in the dropdown menu,

the joint centre of the sphere is active (1) and the sphere is visible, and the joint centre of the invisible ellipsoid is inactive (0).

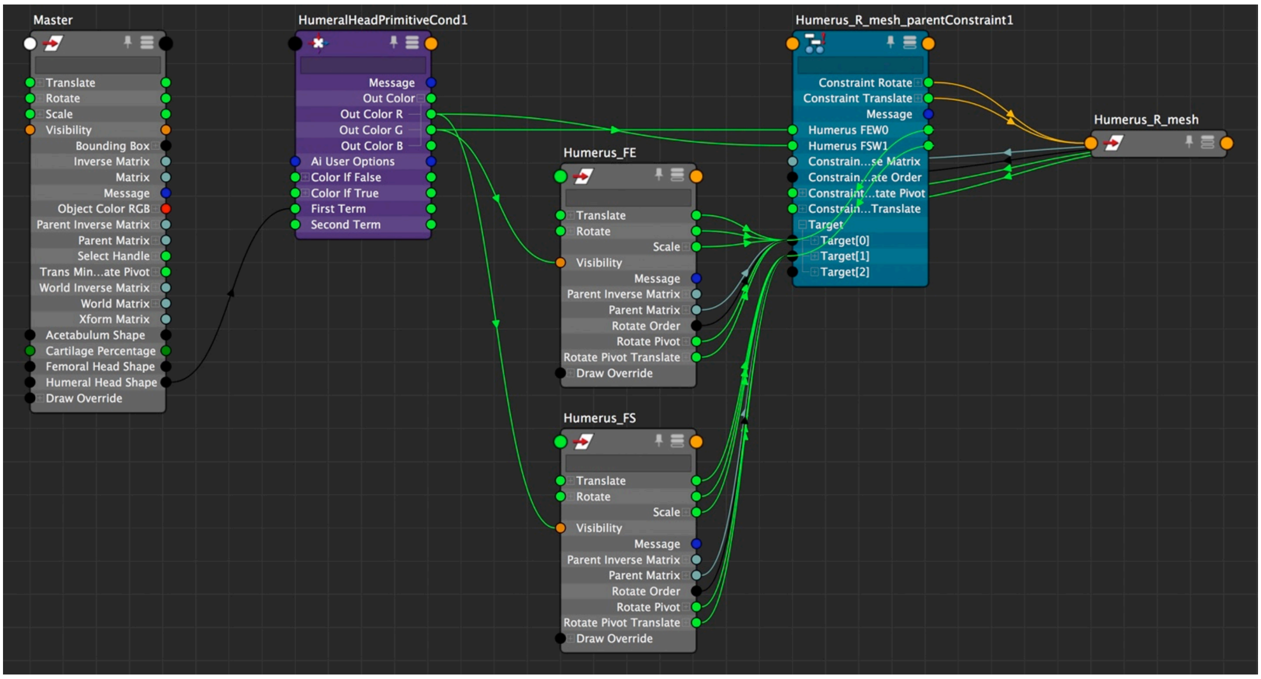

Supplementary Figure S17. Node Editor connections for the primitive shape selector dropdown menu. In this case the setup for the humerus is shown, which is identical to the femur and other bones. Humerus_FE and Humerus_FS nodes are the primitive shapes, the fitted ellipsoid (FE) and the fitted sphere (FS) respectively.

To superimpose the femoral primitive shape over the acetabulum, select the fitted shape of the acetabulum first and then one of the femoral primitive shapes. Click on the box behind *Constrain* > *Point* and make sure that this time the *Maintain offset* box is unchecked and everything set to 0, then click apply. The femoral primitive shape, including the femur, should immediately snap into position in the acetabulum. Repeat this with the other primitive shape on the femoral head. Now when you switch between the primitive shapes in the *Master*, the femur adjusts automatically within the acetabulum.

If there are multiple primitive shapes fitted to the acetabulum, you can repeat the process above and create attribute and dropdown menu to select the fitted shapes in the hip. This is further expandable to accommodate multiple hip morphologies and collision objects, however it is a bit trickier with the Boolean operation, see below.

Boolean specimen selector.

The following approach builds on top of the methodology proposed by Manafzadeh & Padian[10], especially Method S2 in their supplementary material. It explains how two different specimens can be integrated into a single rig and the specimens selected through a dropdown menu in the *Channel Box*. If only a single specimen is to be assessed or if the different specimens are set up independently, this paragraph is irrelevant.

Maya cannot use data arrays, like the vertex coordinates of a 3D mesh, in combination with *conditions* (see above) therefore a s*criptJob* had to be used to enable the dropdown menu. After the BooleanOperation has been created an additional attribute has to be added to the *Master* (see above). Add an *Enum* type attribute called "BooleanSpecimenSelector". The exact *Enum Names* are not relevant, however they should reflect the different specimens for the Boolean operation.

Once the attribute is created, the following MEL script can be used to initialize the BooleanSpecimenSelector *scriptJob*:

```
global proc BooleanSpecimenSelector() {
	int $specimen = `getAttr "Master.SpecimenSelector"`; //attribute in the Master locator
	if ($specimen == 1)
	{
		connectAttr -f Pelvis_01.outMesh polyCBoolOp1.inputPoly[0]; //name "Pelvis_01"
according to first Boolean colliders (Pelvis)
		connectAttr -f Pelvis_01.worldMatrix[0] polyCBoolOp1.inputMat[0];
	}
	else
	{
		connectAttr -f Pelvis_02.outMesh polyCBoolOp1.inputPoly[0]; //name "Pelvis_02"
according to second Boolean colliders (Pelvis)
		connectAttr -f Pelvis_02.worldMatrix[0] polyCBoolOp1.inputMat[0];
	 }
}
int $scriptJobNum = `scriptJob -attributeChange "Master.SpecimenSelector"
"BooleanSpecimenSelector" `;
```

The *scriptJob* gets triggered whenever the *Master.SpecimenSelector* attribute changes and it automatically links the *outMesh* and *worldMatrix* of the corresponding mesh to the *BooleanOperation*.

Unfortunately, *scribtJobs* are global and are therefore executed with every startup of Maya. Thus it has to be stopped (killed) manually before the Maya window is closed, otherwise issues might occur when the *scriptJob* is created for a new simulation.

After creation, every *scriptJob* gets a number assigned, displayed as // Result: 123 //, which gets attributed procedurally and thus might change every time it is created. If you forget the number, *scriptJobs* can be quarried with the following MEL script:

```
scriptJob -listJobs;
```

This will list all currently running *scriptJobs* in the *Script Editor*. Look for the number, followed by: "-attributeChange" "Master.SpecimenSelector" "BooleanSpecimenSelector"

The following MEL script can be used to kill the *BooleanSpecimenSelector scriptJob* and thus remove it completely.

```
scriptJob -kill $jobNum -force; //while $jobNum should be replaced by the number from
above, e.g. 123
```

Alternatively, the *BooleanSpecimenSelector* could also be done with an expression, however this is not recommended and would result in a much longer simulation time. The expression

would check for every single frame, if the *Master.SpecimenSelector* attribute changes, or not, and then relink the *outMesh* and the *worldMatrix* of the respective specimen to the Boolean operation. This results in a lot of unnecessary calculation power used as the attribute is not going to change throughout the simulation and thus needlessly increases the duration of the simulation.

Cartilage slider.

To create a dynamic variable for the slider an integer attribute has to be added to the *Master*. Select the *Master* selected and click on *Modify > Add attributes*. Name it *CartilageSlider* and choose *Integer. Float* would also work however sub percentage resolution is not necessary, as a resolution of 1-percent increments is sufficient. Set the Minimum under *Numeric Attribute Properties* to 0 and the Maximum to 100, thus creating a range from 0 to 100 percent additional cartilage.

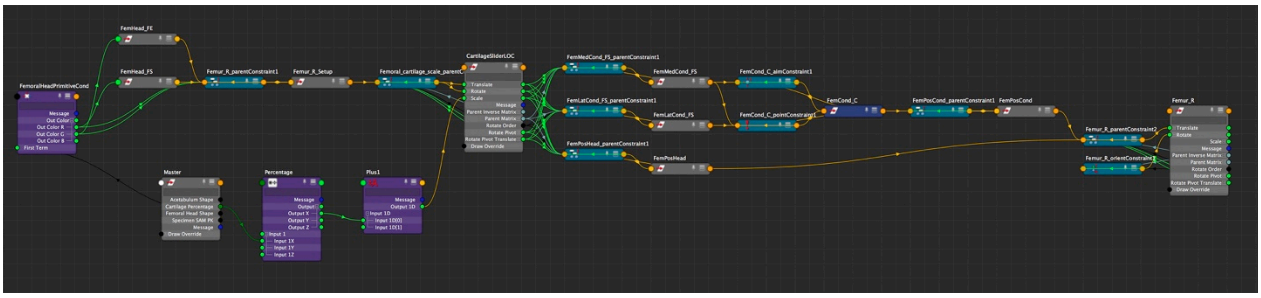

Supplementary Figure S18. Node Editor connections for the cartilage slider. Note that the translation and rotation of the of the *CartilageSliderLOC* are derived from the active primitive shape, while the scale is controlled by the cartilage slider.

In the *Node Editor* create a *multiplyDivide* node and connect the output from the *CartilageSlider* with the *Input 1X* of the newly created node. Set the *Input 2X* to 100 and the *Operation* to *Divide*. Create a *plusMinusAverage* average node and connect the *Output X* of the *multiplyDivide* node with the *Input 1D[0]* attribute of the *plusMinusAverage* node and set the *Input 1D[1]* to 1 and the *Operation* to *Sum*. These simple calculations transform the percentage of the slider in a scale factor for later use (e.g. if the *CartilageSlider* is set to 50, the output value of the *plusMinusAverage* node is going to be 1.5, if it is set to 10, the output is going to be 1.1).

Create a *Locator*, rename it to *CartilageSliderLOC* and *parent constrain* it to the primitive shape (or shapes), dependent on the active shape, as explained in the Primitive shape selector paragraph above. Then connect the Output 1D of the *plusMinusAverage* node with each *Scale* input (*Scale X*, *Scale Y* and *Scale Z*) of the *CartilageSliderLOC* (Supplemetary Fig. S18). Thus when the amount of additional cartilage is increased, the *CartilageSliderLOC* is scaled accordingly. This affects everything downstream that is parented to or under it. When the local scale is increased or decreased, the world position of the parented object changes as they as they are positioned relative to local scale of *CartilageSliderLOC*.

Instead of parenting the femur directly to the primitive shapes, parent it to the primitive shape fitted to the distal condyles, similar to the layout in Supplementary Fig. S18. As the shape fitted to the distal condyle is influenced by the *CartilageSliderLOC*, it enables the cartilage slider to take effect on the femur.

Force Boolean update work-around

We have encountered an issue with the Boolean operation in multiple versions of Maya (Maya 2017, 2018 and 2019 for Windows) on multiple workstations, however, it never occurred in Maya 2017 or Maya 2019 for Mac. The Boolean operation can get stuck at the first frame and does not update with the simulation unless it receives a transformation input. The issue can occur randomly when a simulation is started (stuck on frame 1) or when it is started on one workstation, paused and then continued on another workstation (stuck on the previously paused frame). We are currently unable to replicate this issue again nor could we determine its cause, however, should the issue occur, it can be solved with an additional expression.

After the two meshes have been selected and the Boolean operation performed (see Manafzadeh & Padian[10], supplementary material, Method S2), you can see in the *Outliner* that the meshes have changed into group nodes with a transform node parented under each. Select the node containing the pelvis and create a new expression using the following MEL code in the *Expression Editor*:

```
setAttr "BooleanObject1.translateX" -0.0001; //rename BooleanOject1 according to selected mesh
setAttr "BooleanObject1.translateX" 0.0000; //rename BooleanOject1 according to selected mesh
```

This ensures that for each frame the animation is played, the pelvis receives a translation input, moving it 0.0001 units in negative X direction and setting it back to 0, and thus the Boolean operation does not get stuck after the first collision. It is important, however, that the *Transformations* of the pelvis have been *frozen* (everything set to zero) before executing the expression, otherwise the pelvis gets moved to its null-position, which might not correspond with the desired position for the simulation.

Calculation of joint spacing

To determine the best joint setup for *Euparkeria* and *Crocodylus niloticus* based on several parameters from the fitted primitive shapes, generated with the Shape_Fitter script[9], a short MATLAB script was implemented. With the following script the joint spacing, the radii of the fitted primitive shapes and the distance between their centroids in the articulated hip joint of *C. niloticus* and *Euparkeria* were calculated (Supplementary Table S3 and S4).

```
%% ===== Load data =====
Ace_FP = importdata('IMPORT_FITTED_PRIMITIVE.obj', ' ', 4); %load fitted primitive shape
fitted to the acetabulum
Fem_FP = importdata('IMPORT_FITTED_PRIMITIVE.obj', ' ', 4); %load fitted primitive shape
fitted to the femoral head

%% ===== acetabulum centroid =====
Ace_VTCArray = Ace_FP.data;
Ace_VTCArray(643,:) = [];
Ace_CentroidPos = mean(Ace_VTCArray,1);

%% ===== femoral head centroid =====
Fem_VTCArray = Fem_FP.data;
Fem_VTCArray(643,:) = [];
Fem_CentroidPos = mean(Fem_VTCArray,1);

%% ===== calculate average radius of fitted shapes ====
Ace_rad = sqrt(sum((Ace_VTCArray - Ace_CentroidPOS).^2, 2));
Ace_radAvg = mean(Ace_rad,1);
Fem_rad = sqrt(sum((Fem_VTCArray - Fem_CentroidPOS).^2, 2));
Fem_radAvg = mean(Fem_rad,1);

%% ===== calculate centroid distance and joint spacing ====
CentroidDist = sqrt(sum((Ace_CentroidPOS - Fem_CentroidPOS).^2, 2));
JointSpacingDist = Ace_radAvg - Fem_radAvg + CentroidDist;
```

SUPPLEMENTARY REFERENCES

1. Kambic, R. E., Roberts, T. J. & Gatesy, S. M. Long-axis rotation: a missing degree of freedom in avian bipedal locomotion. *J. Exp. Biol.* **217**, 2770–2782 (2014).

2. Tsai, H. P., Turner, M. L., Manafzadeh, A. R. & Gatesy, S. M. Contrast-enhanced XROMM reveals in vivo soft tissue interactions in the hip of *Alligator mississippiensis*. *J. Anat.* **236**, 288–304 (2019).

3. Parrish, J. M. Locomotor adaptations in the hindlimb and pelvis of the Thecodontia. *Hunteria* **1**, 1–35 (1986).

4. Bonnan, M. F. *et al.* Calcified cartilage shape in archosaur long bones reflects overlying joint shape in stress-bearing elements: Implications for nonavian dinosaur locomotion. *Anat. Rec. Adv. Integr. Anat. Evol. Biol.* **293**, 2044–2055 (2010).

5. Holliday, C. M., Ridgely, R. C., Sedlmayr, J. C. & Witmer, L. M. Cartilaginous epiphyses in extant archosaurs and their implications for reconstructing limb function in dinosaurs. *PLoS One* **5**, e13120 (2010).

6. Otero, A., Allen, V. R., Pol, D. & Hutchinson, J. R. Forelimb muscle and joint actions in Archosauria: insights from *Crocodylus johnstoni* (Pseudosuchia) and *Mussaurus patagonicus* (Sauropodomorpha). *PeerJ* **5**, e3976 (2017).

7. Gatesy, S. M., Baier, D. B., Jenkins, F. A. & Dial, K. P. Scientific rotoscoping: A morphology-based method of 3-D motion analysis and visualization. *J. Exp. Zool. Part A Ecol. Genet. Physiol.* **313A**, 244–261 (2010).

8. Nyakatura, J. A., Andrada, E., Curth, S. & Fischer, M. S. Bridging "Romer's Gap": Limb mechanics of an extant belly-dragging lizard inform debate on tetrapod locomotion during the arly Carboniferous. *Evol. Biol.* **41**, 175–190 (2014).

9. Bishop, P. J., Cuff, A. R. & Hutchinson, J. R. How to build a dinosaur: Musculoskeletal modelling and simulation of locomotor biomechanics in extinct animals. *Paleobiology* (in review).

10. Manafzadeh, A. R. & Padian, K. ROM mapping of ligamentous constraints on avian hip mobility: implications for extinct ornithodirans. *Proc. R. Soc. B Biol. Sci.* **285**, 20180727 (2018).
